# Association between systemic inflammation biomarkers and incident cardiovascular disease in 423,701 individuals: evidence from the UK biobank cohort

**DOI:** 10.1186/s12933-025-02721-9

**Published:** 2025-04-15

**Authors:** Pei Qin, Frederick K. Ho, Carlos A. Celis-Morales, Jill P. Pell

**Affiliations:** 1 School of Health and Wellbeing, University of Glasgow, Clarice Pears Building, 90 Byres Road, Glasgow, G12 8TB UK; 2 Shenzhen Qianhai Shekou Free Trade Zone Hospital, Shenzhen, China; 3 School of Cardiovascular and Metabolic Health, University of Glasgow, Glasgow, UK; 4 Human Performance Lab, Education, Physical Activity and Health Research Unit, University Católica del Maule, Talca, Chile; 5 Centro de Investigación en Medicina de Altura (CEIMA), Universidad Arturo Prat, Iquique, Chile

**Keywords:** Systemic inflammation, CVD, Ischaemic heart disease, Stroke, Heart failure, UK biobank

## Abstract

**Background:**

The associations between systemic inflammation biomarkers and cardiovascular disease (CVD) remain not well explored. This study aimed to investigate associations between different systemic inflammation biomarkers and incident CVD and main CVD subtypes - ischaemic heart disease (IHD), stroke, and heart failure - explore dose–response relationships, and compare their predictive performance.

**Methods:**

This prospective cohort study included 423,701 UK Biobank participants free of CVD at baseline. Baseline neutrophil-to-lymphocyte ratio (NLR), lymphocyte-to-monocyte ratio (LMR), platelet-to-lymphocyte ratio (PLR), systemic immune-inflammation index (SII), and system inflammation response index (SIRI) were derived. Cox-proportional regression models were used to investigate the associations.

**Results:**

NLR, PLR, SII, and SIRI was positively and LMR was negatively associated with all four of the outcomes investigated. The relationships were non-linear for all biomarkers with CVD and were linear for NLR, SII, and SIRI and non-linear for LMR and PLR with IHD, stroke and heart failure. Compared with the more established biomarkers, all four of the novel biomarkers had statistically superior predictive performance for three of the outcomes investigated (CVD, IHD and heart failure) and three of them were superior at predicting stroke. Compared to a model of CVD prediction with classical risk factors (C-index = 0.702), discrimination was improved on the addition of inflammation markers for CVD (C-index change 0.0069, 95% CI 0.0033 to 0.0107), IHD (C-index change 0.0054, 95% CI 0.0013 to 0.0095), and heart failure (C-index change 0.0153, 95% CI 0.0089 to 0.0218).

**Conclusions:**

There were independent and dose–response relationships between the novel systemic inflammation biomarkers and CVD outcomes. Addition of the inflammation biomarkers including novel inflammation biomarkers showed improved discrimination of the traditional risk prediction model. With accumulated evidence, these biomarkers should be considered for inclusion in risk tools and prevention.

**Graphical abstract:**

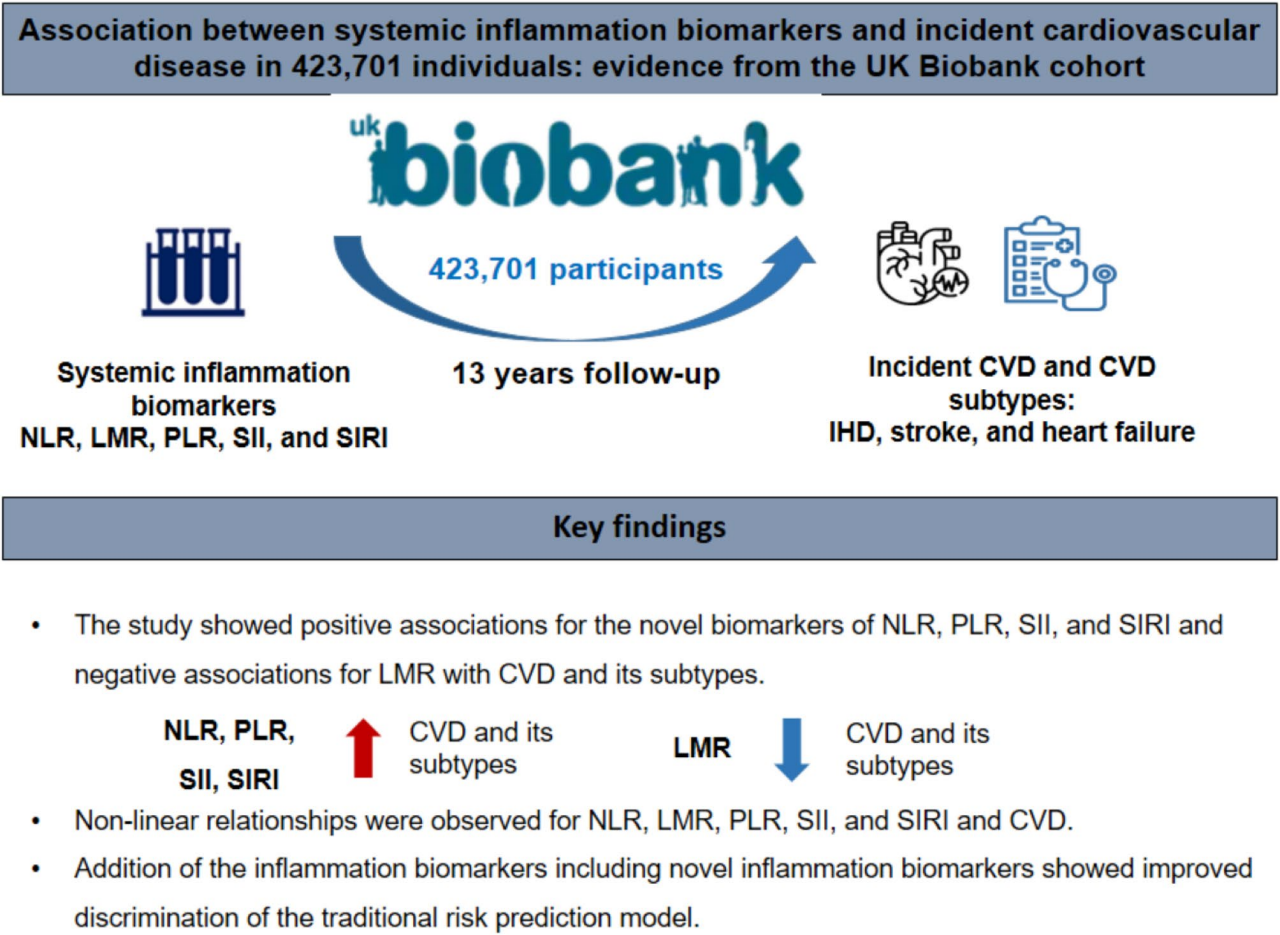

**Supplementary Information:**

The online version contains supplementary material available at 10.1186/s12933-025-02721-9.

## Introduction

Cardiovascular disease (CVD) is a significant global public health problem [1]. The number of prevalent CVD cases worldwide doubled over 30 years, reaching 523 million in 2019 [1]. Given the increasing burden of CVD, it is important to identify factors that could inform prevention, diagnosis, and treatment.

Inflammation has been shown to contribute to the development of a series of chronic diseases [2]. Some novel biomarkers - neutrophil-to-lymphocyte ratio (NLR), platelet-to-lymphocyte-ratio (PLR), lymphocyte-to-monocyte ratio (LMR), systemic immune-inflammation index (SII) and system inflammation response index (SIRI)- were derived from lymphocyte, neutrophil, monocyte and platelet counts and shown to be indicators of systemic inflammation [3], which are worthy to be explored as biomarkers of disease because of they are simple and easily accessible. Evidence is accumulating of the associations of these new biomarkers with cancer [3], dementia [4], cerebrovascular plaques [5], arrhythmias [6], and premature death [7]. Existing studies on the associations between NLR, SII, and SIRI and CVD were small-scale and focused on population sub-groups (8–10) whilst other studies on NLR, LMR, and PLR only explored progression of CVD or its subtypes (11–13). Furthermore, no study has compared the effect estimates of different systemic inflammation biomarkers within the same cohort, and the dose–response relation remains unclear.

To address these knowledge gaps, this study aimed to assess the associations, dose–response relationships and predictive performances of a panel of different systemic inflammation biomarkers (neutrophil count, monocyte count, lymphocyte count, CRP, LMR, NLR, PLR, SII) with incident CVD and CVD subtypes (ischaemic heart disease (IHD), stroke, and heart failure) using UK Biobank data.

## Methods

### Study sample and participants

A prospective cohort study was undertaken using data from UK Biobank, in which approximately 500,000 participants were recruited from the general population via 22 assessment centres across England, Scotland and Wales between 2006 and 2010. All participants were invited to complete a touch screen questionnaire at baseline, have physical measurements taken, and provide biological samples. The detailed protocol is available online (https://www.ukbiobank.ac.uk/media/gnkeyh2q/study-rationale.pdf) and has been described previously [8]. The touch screen questionnaire and other resources are available on the UK Biobank website (http://www.ukbiobank.ac.uk/key-documents/). The study was approved by the North-West Multi-centre Research Ethics Committee [8]. All participants provided written informed consent in accordance with the Declaration of Helsinki.

**Fig. 1:**
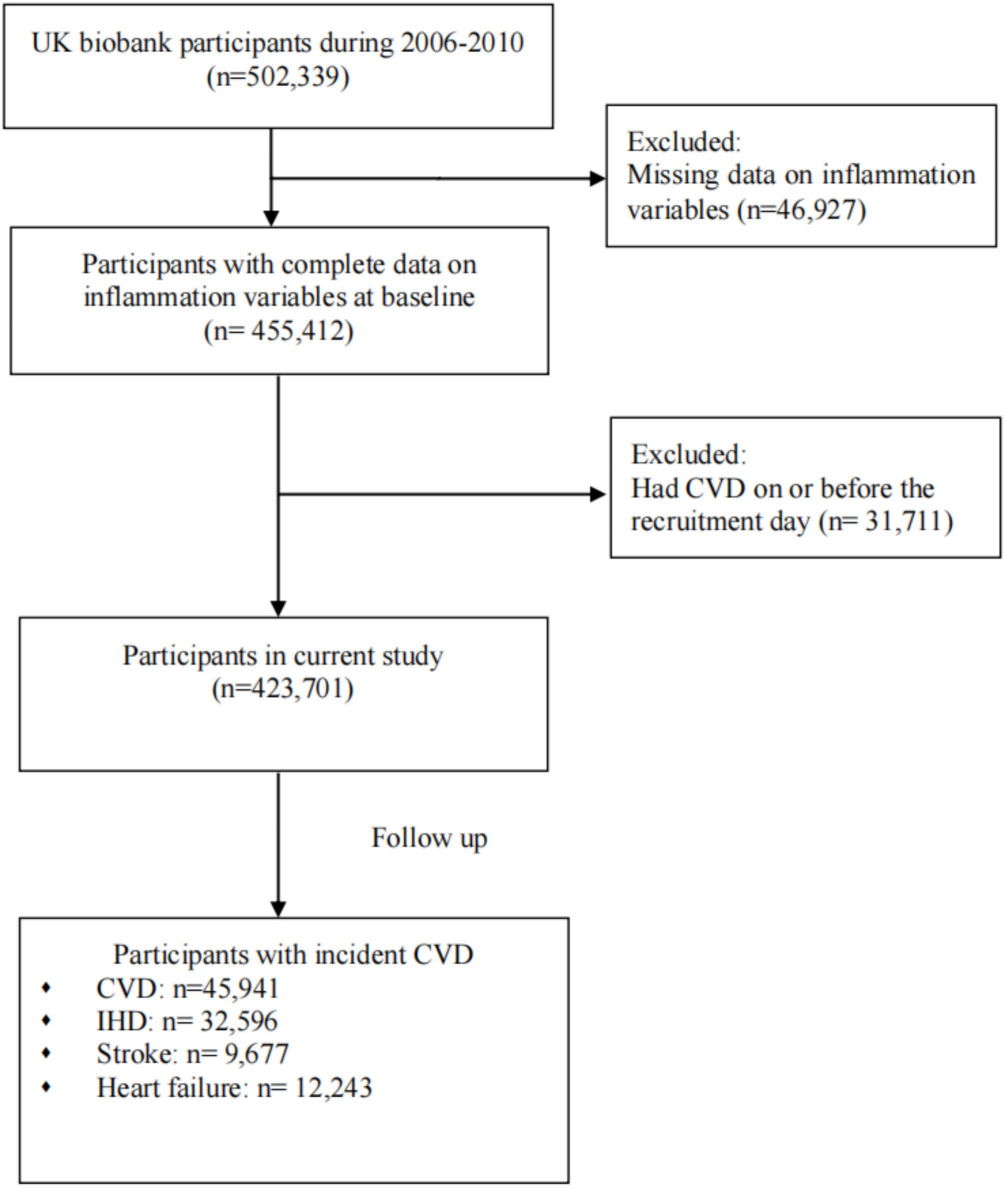
Flowchart of participant inclusion

The following exclusion criteria were applied: (i) participants with missing data on systemic inflammation biomarkers at baseline (*n* = 46,927) and (ii) participants who had CHD, stroke, or heart failure based on the self-report physician diagnosis, hospital admission, day case records and death record data (*n* = 31,711). Hence, 423,701 individuals were included in this study (Fig. 1).

### Systemic inflammation biomarkers

Peripheral blood samples at baseline were analysed at the UK Biobank central laboratory within 24 h of drawing blood using a Beckman Coulter LH750 Hematology Analyzer. Baseline count data for neutrophils, monocytes, lymphocytes, and platelets were obtained and used to derive five additional systemic inflammation biomarkers: NLR (calculated as neutrophil/lymphocyte), LMR (lymphocyte/monocyte), PLR (platelet/lymphocyte), SII (neutrophils ×platelets/lymphocyte), and SIRI (monocyte×neutrophil/lymphocyte). Serum C-reactive protein (CRP) was measured with the immunoturbidimetric high-sensitivity assay using a Beckman Coulter AU5800. Details of data processing and the quality check procedure are available on the website of UK Biobank [9, 10].

### Cardiovascular disease

The primary outcome was CVD and the secondary outcomes were three CVD sub-types: IHD, stroke, and heart failure. The outcomes were ascertained from the disease codes recorded on linked hospital admission and death records. Using International Statistical Classification of Diseases and Related Health Problems, Tenth Revision (ICD-10) codes, primary/secondary CVD and their subtypes were denoted, where CVD was defined as I20-I25, I60-I64, I50, I42.0, I42.6, I42.7, I42.9, I11.0, IHD as I20–25, stroke as I60–64, and heart failure as I11.0, I42.0, I42.6–42.7, I42.9 and I50. These codes were used to reduce the risk of missing diagnosis. Notably, Stroke was defined as a composite outcome including both ischaemic (I63) and haemorrhagic stroke (I60–I62), as well as unspecified stroke (I64), as chronic inflammation was common risk factor of both subtypes. CVD outcomes were defined by combining non-fatal and fatal CVD events and the endpoint time is ascertained as the first diagnosis for CVD events or the occurrence of death, or the censoring date, whichever occurred first. For the occurrence of CVD, events were censored at first CVD event of CHD, stroke, or heart failure, whichever came first. The occurrence of subtypes of cardiovascular outcomes was censored for the first date of events of each subtype of CVD outcome.

### Covariates

The covariates included in this study as potential confounders were: age at baseline assessment (calculated from date of birth and date of baseline assessment), sex (male or female), ethnicity (white, South Asian, or other), Townsend deprivation index (analysed as tertiles: low, moderate, and high), smoking status (never, former, current), alcohol drinking (average weekly units) [11], physical activity (low, moderate, high) [12], sedentary behavior (hours per day), fruit and vegetable intake (portions per day), sleep duration (1–6, 7–8, or ≥ 9 h/day), red meat intake (0, 0–2, > 2 times/week) [13], processed meat intake (0, 0–2, > 2 times/week) [13], body mass index (BMI), waist circumference (WC), blood pressure, serum total cholesterol, high-density lipoprotein (HDL) cholesterol, and glycated haemoglobin (HbA1c), and number of long-term conditions (LTCs) (0, 1, or ≥ 2) [14]. Average weekly units of alcohol consumed were calculated based on the questions regarding whether consumed alcohol, frequency of consumption and the typical weekly/monthly consumption of measures of different types of alcoholic drinks. These measures were converted into an estimate of their average number of UK units of alcohol (1 unit = 8 g of ethanol) consumed weekly [11]. Forty long-term conditions (LTCs) diagnosed by doctors and self-reported by participants at baseline were summed and categorized into: 0, 1, or ≥ 2, which were adapted from 43 LTCs by excluding coronary heart disease, heart failure, or stroke (Supplemental Table 1) [14]. The measurement of covariates has been described in previous studies [15]. Additional details about these measurements can be found on the UK Biobank website (https://www.ukbiobank.ac.uk/).

### Statistical analyses

Baseline characteristics of the study population were described as frequency (percentages), mean (standard deviation, SD), or median (interquartile range, IQR) where appropriate. Differences in baseline characteristics across groups by the outcomes and missing status on the inflammation biomarker data were assessed using analysis of variance (ANOVA), Mann–Whitney U, and χ^2^ tests, respectively.

Cox proportional hazard models were utilized to calculate the hazard ratios (HRs) and 95% confidence intervals (CIs) for the associations between systemic inflammation biomarkers and incident outcomes, because it is a powerful method to focus on time-to-event outcomes and account for both censored data and multiple covariates [16]. We used the Schoenfeld Residuals test and log–log survival plots to evaluate the proportional hazard assumption (no violation was observed). Confounders in the models were identified based on a priori knowledge from previous studies [17, 18] and principles of confounders selection [19]; a minimal sufficient adjustment set was identified by a directed acyclic graph (DAG) using the DAGitty online tool (http://www.dagitty.net), as shown in Supplemental Fig. 1. Three models were adopted: Model 1 adjusted for age, sex, Townsend deprivation index, and ethnicity; Model 2 additionally adjusted for smoking status, alcohol consumption, physical activity, total sedentary time, sleep duration, and fruit and vegetable intake; Model 3 additionally adjusted for total cholesterol, HDL cholesterol, SBP, HbA1c, and number of LTCs. Trend analyses were also undertaken of the risk of incident outcomes per 1-SD increment in systemic inflammation biomarkers. A model that additionally adjusted for other inflammatory biomarkers when exploring each exposure and incident CVD was also adopted to explore if the inflammatory biomarker is independent of other inflammatory biomarkers. We used restricted cubic splines with 3 knots placed at the 10th, 50th, and 90th percentiles to assess the potential non-linear relationship between systemic inflammation biomarkers expressed as continuous exposure variables with incident CVD, after removing 1‰ outliers of the biomarkers. The Harrell’s concordance statistic (C-statistic) was also calculated to estimate the predictive performance of the inflammatory biomarkers for the outcome. *P*-values were calculated for the differences between the C-statistics of these inflammatory biomarkers using the DeLong test [20]. The change in Harrell’s C-index (C-statistic) for each outcomes was further used to assess the effects of the addition of different systemic inflammation biomarkers to the SCORE2 Cox proportional hazards model risk score: age, sex, systolic blood pressure, smoking, total cholesterol level, HDL cholesterol level [21].

A series of sensitivity analyses were performed: (i) excluding participants with incident CVD in the first two years (*n* = 4,275); (ii) additionally adjusting for insulin, and antihypertensive and cholesterol-lowering medications; (iii) excluding participants who have statin (*n* = 7,637); (iv) adjusting for Downtown deprivation index as continuous variable instead of categorical variable; (v) imputing data of the missing covariates at baseline, where we imputed missing data using the multiple imputation by chained equations approach [22] and results were pooled across 10 imputed data sets according to Rubin’s rules. The analyses were conducted using R, version 4.3.2 (R Foundation for Statistical Computing) statistical packages. Two-tailed *P*-values < 0.05 were considered to indicate significance.

## Results

### Participant baseline characteristics

A total of 423,701 participants were included in the study. Their mean age was 56 years (SD 9.3; range 38–73 years), 54.7% were women, and 96.7% were of white ethnicity (Table 1). Over a median follow-up period of 13.4 (IQR 13.0–14.8) years, 45,941 individuals developed CVD (8.8%): 32,596 developed IHD, 9,677 stroke, 12,243 heart failure; of them, 37,976 developed only one subtype, 7,355 developed two subtypes, and 610 developed 3 subtypes. Compared to those without incident CVD, participants with CVD were more likely to be male, current smokers, live in deprived areas, and be less physically active and spend more time sedentary, consume more alcohol, red and processed meat and fewer vegetables and fruit, and have higher WC and BMI, and more LTCs. Participants who developed CVD had higher counts of neutrophils, monocytes, and lymphocytes, higher values of NLR, PLR, SII, CRP, but lower values of LMR (Table 1). A similar pattern was observed when stratified by CVD subtypes (Supplementary Table 2). Supplementary Table 3 shows these characteristics by groups with and without missing data in inflammation biomarkers. There were discernible differences in the proportions of male participants, category of deprivation index, ethnic groups, lifestyle, and biological markers; however, the Cohen’d showed the small between-group differences (data not shown).

**Table 1:** Baseline characteristics of participants in the UK biobank

	Overall	Incident CVD at follow up		*P*
		No	Yes	
N	423,701	377,760	45,941	
Age (years), mean (SD)	56.17 (8.09)	55.68 (8.08)	60.20 (6.95)	< 0.001
Men, n (%)	188,023 (44.4)	160,812 (42.6)	27,211 (59.2)	< 0.001
Deprivation index, n (%)				< 0.001
Low	143,835 (34.0)	129,428 (34.3)	14,407 (31.4)	
Moderate	142,016 (33.6)	126,798 (33.6)	15,218 (33.2)	
High	137,317 (32.4)	121,050 (32.1)	16,267 (35.4)	
Ethnicity (%)				< 0.001
White	399,744 (96.9)	356,339 (96.9)	43,405 (96.7)	
South Asia	6361 ( 1.5)	5422 ( 1.5)	939 ( 2.1)	
Other	6505 ( 1.6)	5978 ( 1.6)	527 ( 1.2)	
Smoking status, n (%)				< 0.001
Never	235,367 (55.8)	214,065 (56.9)	21,302 (46.7)	
Previous	142,649 (33.8)	124,703 (33.2)	17,946 (39.3)	
Current	43,668 (10.4)	37,264 ( 9.9)	6404 (14.0)	
Alcohol (weekly units), median (IQR)	10.50 (2.90, 22.50)	10.5 (3.00, 22.20)	11.4 (1.50, 25.20)	< 0.001
Physical activity, n (%)				< 0.001
Low	63,327 (18.4)	55,942 (18.2)	7385 (20.3)	
Moderate	140,179 (40.8)	125,800 (41.0)	14,379 (39.6)	
High	139,856 (40.7)	125,321 (40.8)	14,535 (40.0)	
Sedentary time (hours), mean (SD)	4.47 (2.57)	4.42 (2.55)	4.90 (2.70)	< 0.001
Fruit and vegetable intake (portion per day), mean (SD)	4.11 (2.43)	4.12 (2.42)	4.03 (2.53)	< 0.001
Processed meat intake (times/week), n (%)				< 0.001
0	39,765 ( 9.4)	36,334 ( 9.7)	3431 ( 7.5)	
0–2	252,519 (59.8)	226,266 (60.1)	26,253 (57.4)	
> 2	129,943 (30.8)	113,881 (30.2)	16,062 (35.1)	
Red meat intake (times/week), n (%)				< 0.001
0	28,970 ( 6.9)	26,715 ( 7.2)	2255 ( 5.0)	
0–2	256,928 (61.4)	230,111 (61.6)	26,817 (59.4)	
> 2	132,544 (31.7)	116,442 (31.2)	16,102 (35.6)	
Sleep duration (hours/day), n (%)				< 0.001
1–6	102,651 (24.4)	90,271 (24.1)	12,380 (27.2)	
7–8	287,498 (68.3)	258,615 (68.9)	28,883 (63.5)	
≥9	30,546 ( 7.3)	26,314 ( 7.0)	4232 ( 9.3)	
BMI (kg/m^2^), mean (SD)	27.28 (4.72)	27.12 (4.65)	28.56 (5.07)	< 0.001
WC (cm), mean (SD)	89.74 (13.28)	89.09 (13.09)	95.07 (13.61)	< 0.001
HDL (mmol/L), mean (SD)	1.46 (0.38)	1.47 (0.38)	1.36 (0.37)	< 0.001
TG (mmol/L), mean (SD)	1.73 (1.01)	1.71 (1.00)	1.96 (1.10)	< 0.001
LDL (mmol/L), mean (SD)	3.61 (0.85)	3.61 (0.84)	3.61 (0.91)	0.451
Total Cholesterol (mmol/L), mean (SD)	5.76 (1.11)	5.77 (1.10)	5.71 (1.20)	< 0.001
HbA1c (mmol/mol), mean (SD)	35.84 (6.35)	35.60 (5.98)	37.83 (8.57)	< 0.001
SBP (mmHg), mean (SD)	137.69 (18.58)	136.90 (18.39)	144.16 (18.84)	< 0.001
DBP (mmHg), mean (SD)	82.36 (10.08)	82.14 (10.03)	84.12 (10.30)	< 0.001
Number of long-term conditions, n (%)				< 0.001
0	156,056 (36.8)	145,606 (38.5)	10,450 (22.7)	
1	143,499 (33.9)	128,640 (34.1)	14,859 (32.3)	
≥ 2	124,146 (29.3)	103,514 (27.4)	20,632 (44.9)	
Diabetes, n (%)	17,843 ( 4.2)	13,501 ( 3.6)	4342 ( 9.5)	< 0.001
Hypertension, n (%)	103,370 (24.4)	84,778 (22.4)	18,592 (40.5)	< 0.001
Hyperlipidemia, n (%)	44,241 (10.4)	36,156 ( 9.6)	8085 (17.6)	< 0.001
Neutrophil count (10^9 cells/L), mean (SD)	4.19 (1.40)	4.16 (1.38)	4.44 (1.50)	< 0.001
Monocyte count (10^9 cells/L), mean (SD)	0.47 (0.28)	0.47 (0.28)	0.51 (0.28)	< 0.001
Lymphocyte count (10^9 cells/L), mean (SD)	1.96 (1.16)	1.96 (1.12)	2.01 (1.44)	< 0.001
NLR, mean (SD)	2.34 (1.19)	2.32 (1.17)	2.48 (1.36)	< 0.001
LMR, mean (SD)	4.66 (4.25)	4.68 (3.77)	4.43 (3.03)	< 0.001
PLR, mean (SD)	142.46 (60.24)	142.73 (59.72)	140.21 (64.36)	< 0.001
SII, mean (SD)	596.68 (354.23)	593.09 (345.60)	626.19 (417.31)	< 0.001
SIRI, median (IQR)	0.94 (0.67,1.33)	0.93 (0.66, 1.31)	1.06 (0.74, 1.51)	< 0.001
CRP (mg/L), median (IQR)	1.31 (0.65, 2.72)	1.27 (0.63, 2.62)	1.74 (0.87, 3.55)	< 0.001

Ref, reference group; BMI, body mass index; CI, confidence interval; CRP, C-reactive protein; CVD, cardiovascular disease; DBP, diastolic blood pressure; HbA1c, glycated haemoglobin; HDL, high-density lipoprotein cholesterol; IQR, interquartile range; LDL, low-density lipoprotein; LMR, Lymphocyte-to monocyte ratio; NLR, Neutrophil-to-lymphocyte ratio; PLR, Platelet-to-lymphocyte ratio; SBP, systolic blood pressure; SII, systemic immune inflammation index; SD, standard deviation; SIRI, system inflammation response index; WC, waist circumference

### Systemic inflammation biomarkers and cardiovascular disease

**Fig. 2:**
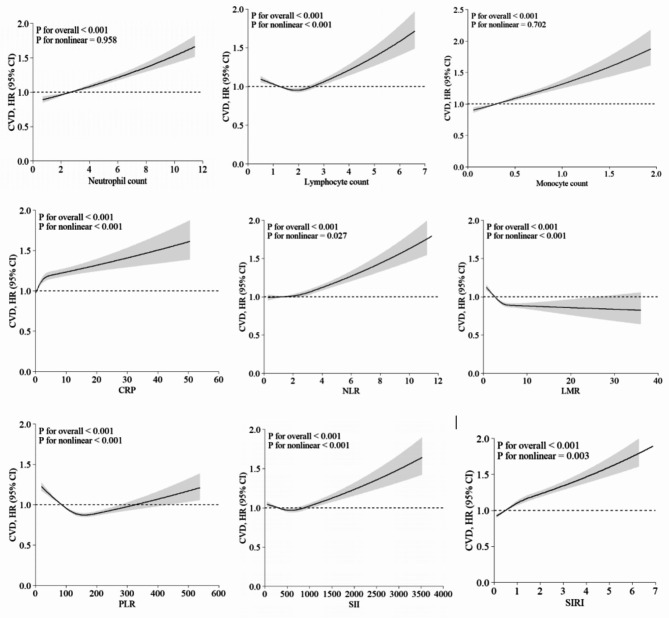
Dose–response associations of systemic inflammation biomarkers and incident CVD. Restricted cubic splines were used to present the associations. The model was adjusted for age, sex, Townsend deprivation index, ethnicity, smoking status, weekly units of alcohol use, sleep duration, fruit and vegetable intake, processed meat intake, red meat intake, physical activity, total sedentary time, number of long-term conditions, HDL, total cholesterol, SBP, HbA1c, BMI, and WC. BMI, body mass index; CI, confidence interval; HDL, high-density lipoprotein cholesterol; HR, hazard ratio; SBP, systolic blood pressure; WC, waist circumference

After adjustment for all potential confounders, the risk of CVD was significantly higher in people with higher neutrophil count, monocyte count, CRP, NLR and SII. No significant association was found for lymphocyte count, while a negative association was found for LMR (Table 2). The relationship of each inflammatory biomarker was independent of the other inflammatory biomarkers (Supplementary Table 4). Non-linear relationships were observed for lymphocyte count (*P*_non−linearity_ < 0.0.001), NLR (*P*_non−linearity_ = 0.027), LMR (*P*_non−linearity_ < 0.001), PLR (*P*_non−linearity_ < 0.001), SII (*P*_non−linearity_ = 0.005), and SIRI (*P*_non−linearity_ = 0.003) (Fig. 2), with lymphocyte count, PLR, and SII showing U-shaped relationships where both lower and higher levels were associated with an increased risk of CVD. There was no evidence of a non-linear relation for Neutrophil count (*P*_non−linearity_ = 0.571), monocyte count (*P*_non−linearity_ = 0.076), and CRP (*P*_non−linearity_ = 0.702), with risk increased by 7%, 4%, and 4% per SD increase, respectively.

**Table 2:** Association of systemic inflammation indicators and incident CVD and subtypes

Outcome	Exposure	Model 1	Model 2	Model 3
		HR (95% CI)	HR (95% CI)	HR (95% CI)
CVD	Neutrophil count			
	Q1	Ref.	Ref.	Ref.
	Q2	1.12 (1.09–1.15)	1.09 (1.06–1.13)	1.05 (1.01–1.09)
	Q3	1.24 (1.21–1.28)	1.19 (1.15–1.23)	1.07 (1.03–1.11)
	Q4	1.51 (1.47–1.55)	1.37 (1.33–1.42)	1.17 (1.13–1.22)
	Per 1-SD increase	1.13 (1.12–1.14)	1.12 (1.11–1.12)	1.07 (1.06–1.08)
	Monocyte count			
	Q1	Ref.	Ref.	Ref.
	Q2	1.07 (1.04–1.1)	1.06 (1.02–1.1)	1.03 (0.99–1.08)
	Q3	1.14 (1.11–1.17)	1.12 (1.08–1.16)	1.05 (1.01–1.09)
	Q4	1.35 (1.31–1.39)	1.29 (1.24–1.33)	1.14 (1.10–1.19)
	Per 1-SD increase	1.01 (1.01–1.01)	1.01 (1.01–1.01)	1.04 (1.03–1.05)
	Lymphocyte count			
	Q1	Ref.	Ref.	Ref.
	Q2	0.99 (0.96–1.02)	0.98 (0.94–1.01)	0.95 (0.91–0.98)
	Q3	1.04 (1.02–1.07)	1.01 (0.97–1.04)	0.94 (0.90–0.97)
	Q4	1.23 (1.20–1.26)	1.14 (1.10–1.17)	1.01 (0.97–1.04)
	Per 1-SD increase	1.02 (1.02–1.03)	1.02 (1.01–1.02)	1.00 (0.99–1.01)
	CRP			
	Q1	Ref.	Ref.	Ref.
	Q2	1.21 (1.17–1.25)	1.16 (1.12–1.20)	1.03 (0.99–1.07)
	Q3	1.42 (1.38–1.46)	1.34 (1.30–1.39)	1.10 (1.06–1.15)
	Q4	1.81 (1.76–1.86)	1.63 (1.57–1.68)	1.20 (1.16–1.25)
	Per 1-SD increase	1.10 (1.10–1.11)	1.09 (1.08–1.1)	1.04 (1.03–1.06)
	LMR			
	Q1	Ref.	Ref.	Ref.
	Q2	0.95 (0.93–0.98)	0.95 (0.92–0.98)	0.97 (0.93–1.00)
	Q3	0.91 (0.88–0.94)	0.90 (0.87–0.93)	0.91 (0.87–0.94)
	Q4	0.93 (0.90–0.96)	0.91 (0.87–0.94)	0.91 (0.87–0.94)
	Per 1-SD increase	1.00 (0.99–1.01)	0.99 (0.98–1.01)	0.99 (0.97–1.01)
	NLR			
	Q1	Ref.	Ref.	Ref.
	Q2	1.00 (0.97–1.03)	1.01 (0.97–1.05)	1.01 (0.97–1.04)
	Q3	1.03 (1.01–1.06)	1.03 (0.99–1.06)	1.00 (0.96–1.04)
	Q4	1.17 (1.14–1.2)	1.16 (1.13–1.20)	1.13 (1.09–1.17)
	Per 1-SD increase	1.04 (1.04–1.05)	1.05 (1.05–1.06)	1.04 (1.04–1.05)
	PLR			
	Q1	Ref.	Ref.	Ref.
	Q2	0.89 (0.87–0.92)	0.92 (0.89–0.95)	0.95 (0.92–0.99)
	Q3	0.86 (0.84–0.89)	0.90 (0.87–0.93)	0.96 (0.92–0.99)
	Q4	0.88 (0.85–0.90)	0.92 (0.89–0.95)	0.99 (0.95–1.02)
	Per 1-SD increase	0.98 (0.97–0.99)	1.00 (0.99–1.01)	1.02 (1.01–1.03)
	SII			
	Q1	Ref.	Ref.	Ref.
	Q2	1.01 (0.98–1.04)	1.01 (0.97–1.04)	1.00 (0.96–1.04)
	Q3	1.05 (1.02–1.08)	1.04 (1.00–1.07)	1.01 (0.97–1.05)
	Q4	1.18 (1.15–1.21)	1.15 (1.11–1.18)	1.09 (1.05–1.13)
	Per 1-SD increase	1.05 (1.05–1.06)	1.05 (1.05–1.06)	1.04 (1.04–1.05)
	SIRI			
	Q1	Ref.	Ref.	Ref.
	Q2	1.09 (1.06–1.12)	1.08 (1.04–1.12)	1.04 (1.00–1.09)
	Q3	1.21 (1.18–1.25)	1.19 (1.15–1.23)	1.12 (1.07–1.16)
	Q4	1.37 (1.33–1.41)	1.33 (1.29–1.38)	1.21 (1.16–1.26)
	Per 1-SD increase	1.03 (1.02–1.03)	1.02 (1.02–1.03)	1.02 (1.01–1.02)
IHD	Neutrophil count			
	Q1	Ref.	Ref.	Ref.
	Q2	1.16 (1.12–1.20)	1.14 (1.10–1.19)	1.09 (1.05–1.14)
	Q3	1.28 (1.24–1.32)	1.25 (1.20–1.30)	1.11 (1.06–1.16)
	Q4	1.52 (1.48–1.57)	1.43 (1.37–1.48)	1.19 (1.14–1.25)
	Per 1-SD increase	1.13 (1.12–1.13)	1.11 (1.10–1.13)	1.07 (1.05–1.08)
	Monocyte count			
	Q1	Ref.	Ref.	Ref.
	Q2	1.08 (1.04–1.12)	1.07 (1.03–1.12)	1.04 (0.99–1.09)
	Q3	1.16 (1.12–1.20)	1.14 (1.09–1.18)	1.06 (1.01–1.10)
	Q4	1.34 (1.30–1.38)	1.29 (1.24–1.34)	1.13 (1.08–1.18)
	Per 1-SD increase	1.01 (1.01–1.01)	1.01 (1.01–1.01)	1.03 (1.02–1.04)
	Lymphocyte count			
	Q1	Ref.	Ref.	Ref.
	Q2	1.03 (1.00–1.06)	1.01 (0.98–1.05)	0.97 (0.94–1.02)
	Q3	1.12 (1.08–1.15)	1.08 (1.04–1.12)	0.99 (0.94–1.03)
	Q4	1.31 (1.27–1.35)	1.23 (1.19–1.28)	1.05 (1.01–1.10)
	Per 1-SD increase	1.02 (1.02–1.03)	1.02 (1.01–1.03)	1.01 (1.00–1.02)
	CRP			
	Q1	Ref.	Ref.	Ref.
	Q2	1.24 (1.20–1.29)	1.20 (1.15–1.25)	1.05 (1.00–1.10)
	Q3	1.48 (1.43–1.53)	1.42 (1.36–1.47)	1.12 (1.07–1.17)
	Q4	1.83 (1.77–1.89)	1.69 (1.62–1.76)	1.19 (1.14–1.25)
	Per 1-SD increase	1.10 (1.09–1.10)	1.08 (1.07–1.09)	1.03 (1.02–1.04)
	LMR			
	Q1	Ref.	Ref.	Ref.
	Q2	0.98 (0.95–1.01)	0.98 (0.94–1.01)	0.98 (0.94–1.02)
	Q3	0.96 (0.92–0.99)	0.95 (0.91–0.99)	0.94 (0.90–0.98)
	Q4	1.00 (0.96–1.03)	0.97 (0.93–1.01)	0.95 (0.90–0.99)
	Per 1-SD increase	1.01 (1.00–1.02)	1.01 (1.00–1.02)	1.00 (0.99–1.02)
	NLR			
	Q1	Ref.	Ref.	Ref.
	Q2	1.01 (0.98–1.04)	1.02 (0.98–1.06)	1.01 (0.97–1.06)
	Q3	1.01 (0.98–1.05)	1.02 (0.98–1.06)	1.00 (0.95–1.04)
	Q4	1.12 (1.09–1.16)	1.12 (1.08–1.17)	1.10 (1.06–1.15)
	Per 1-SD increase	1.03 (1.03–1.04)	1.04 (1.03–1.05)	1.04 (1.03–1.05)
	PLR			
	Q1	Ref.	Ref.	Ref.
	Q2	0.90 (0.86–0.92)	0.91 (0.88–0.95)	0.96 (0.91–0.99)
	Q3	0.85 (0.82–0.87)	0.87 (0.84–0.91)	0.95 (0.91–0.99)
	Q4	0.83 (0.80–0.86)	0.87 (0.83–0.90)	0.95 (0.90–0.99)
	Per 1-SD increase	0.95 (0.94–0.96)	0.97 (0.96–0.98)	1.00 (0.98–1.01)
	SII			
	Q1	Ref.	Ref.	Ref.
	Q2	1.00 (0.97–1.04)	1.00 (0.96–1.04)	0.99 (0.95–1.04)
	Q3	1.03 (1.00–1.07)	1.03 (0.99–1.07)	1.01 (0.96–1.05)
	Q4	1.14 (1.11–1.18)	1.12 (1.08–1.16)	1.06 (1.02–1.11)
	Per 1-SD increase	1.04 (1.04–1.05)	1.04 (1.03–1.05)	1.03 (1.02–1.04)
	SIRI			
	Q1	Ref.	Ref.	Ref.
	Q2	1.08 (1.04–1.12)	1.07 (1.02–1.12)	1.03 (0.98–1.08)
	Q3	1.19 (1.15–1.23)	1.17 (1.12–1.22)	1.09 (1.04–1.15)
	Q4	1.30 (1.25–1.35)	1.26 (1.21–1.32)	1.15 (1.10–1.20)
	Per 1-SD increase	1.02 (1.02–1.03)	1.02 (1.01–1.02)	1.01 (1.01–1.02)
Stroke	Neutrophil count			
	Q1	Ref.	Ref.	Ref.
	Q2	1.01 (0.95–1.08)	0.98 (0.91–1.05)	0.96 (0.88–1.04)
	Q3	1.11 (1.05–1.18)	1.06 (0.98–1.13)	0.97 (0.89–1.05)
	Q4	1.32 (1.25–1.40)	1.22 (1.14–1.30)	1.09 (1.01–1.18)
	Per 1-SD increase	1.11 (1.09–1.13)	1.09 (1.06–1.11)	1.06 (1.03–1.09)
	Monocyte count			
	Q1	Ref.	Ref.	Ref.
	Q2	1.05 (0.99–1.12)	1.02 (0.94–1.09)	1.01 (0.93–1.10)
	Q3	1.14 (1.07–1.21)	1.12 (1.04–1.20)	1.07 (0.98–1.16)
	Q4	1.30 (1.22–1.37)	1.25 (1.17–1.34)	1.15 (1.06–1.25)
	Per 1-SD increase	1.01 (1.01–1.02)	1.01 (1.01–1.02)	1.04 (1.02–1.05)
	Lymphocyte count			
	Q1	Ref.	Ref.	Ref.
	Q2	0.98 (0.93–1.04)	0.97 (0.91–1.03)	0.95 (0.88–1.02)
	Q3	0.96 (0.90–1.02)	0.95 (0.88–1.01)	0.91 (0.84–0.99)
	Q4	1.16 (1.10–1.23)	1.07 (0.99–1.14)	1.02 (0.94–1.10)
	Per 1-SD increase	1.02 (1.01–1.03)	1.00 (0.98–1.02)	0.99 (0.96–1.02)
	CRP			
	Q1	Ref.	Ref.	Ref.
	Q2	1.12 (1.05–1.19)	1.07 (0.99–1.15)	0.99 (0.90–1.07)
	Q3	1.19 (1.12–1.27)	1.13 (1.05–1.22)	0.99 (0.91–1.08)
	Q4	1.46 (1.38–1.55)	1.38 (1.28–1.48)	1.14 (1.05–1.25)
	Per 1-SD increase	1.07 (1.05–1.08)	1.06 (1.04–1.08)	1.03 (1.00–1.05)
	LMR			
	Q1	Ref.	Ref.	Ref.
	Q2	0.94 (0.89–0.99)	0.93 (0.87–0.99)	0.95 (0.88–1.02)
	Q3	0.88 (0.83–0.93)	0.87 (0.81–0.93)	0.89 (0.82–0.96)
	Q4	0.90 (0.84–0.95)	0.86 (0.8–0.92)	0.89 (0.82–0.97)
	Per 1-SD increase	0.95 (0.92–0.99)	0.91 (0.87–0.95)	0.93 (0.88–0.98)
	NLR			
	Q1	Ref.	Ref.	Ref.
	Q2	0.94 (0.89–1.00)	0.97 (0.90–1.03)	0.95 (0.88–1.03)
	Q3	1.00 (0.94–1.06)	0.99 (0.92–1.06)	0.99 (0.91–1.07)
	Q4	1.11 (1.05–1.17)	1.11 (1.03–1.18)	1.06 (0.97–1.14)
	Per 1-SD increase	1.04 (1.03–1.05)	1.05 (1.03–1.07)	1.04 (1.02–1.06)
	PLR			
	Q1	Ref.	Ref.	Ref.
	Q2	0.89 (0.83–0.94)	0.93 (0.86–0.99)	0.95 (0.88–1.03)
	Q3	0.89 (0.83–0.94)	0.93 (0.86–0.99)	0.94 (0.86–1.01)
	Q4	0.97 (0.92–1.03)	1.04 (0.97–1.11)	1.06 (0.98–1.14)
	Per 1-SD increase	1.02 (1.00–1.04)	1.04 (1.02–1.05)	1.03 (1.02–1.05)
	SII			
	Q1	Ref.	Ref.	Ref.
	Q2	1.01 (0.94–1.07)	1.01 (0.94–1.09)	1.02 (0.94–1.10)
	Q3	1.05 (0.99–1.12)	1.05 (0.98–1.13)	1.03 (0.95–1.11)
	Q4	1.18 (1.11–1.25)	1.17 (1.10–1.25)	1.10 (1.02–1.19)
	Per 1-SD increase	1.05 (1.04–1.07)	1.06 (1.04–1.07)	1.05 (1.03–1.06)
	SIRI			
	Q1	Ref.	Ref.	Ref.
	Q2	1.02 (0.95–1.08)	1.00 (0.93–1.08)	0.98 (0.89–1.06)
	Q3	1.14 (1.08–1.22)	1.12 (1.04–1.21)	1.06 (0.98–1.15)
	Q4	1.28 (1.21–1.36)	1.25 (1.16–1.34)	1.15 (1.06–1.25)
	Per 1-SD increase	1.02 (1.01–1.03)	1.02 (1.01–1.03)	1.02 (1.00–1.03)
Heart failure	Neutrophil count			
	Q1	Ref.	Ref.	Ref.
	Q2	1.15 (1.08–1.22)	1.08 (1.00–1.15)	1.06 (0.98–1.15)
	Q3	1.35 (1.28–1.43)	1.22 (1.14–1.30)	1.11 (1.03–1.20)
	Q4	1.89 (1.80–2.00)	1.55 (1.45–1.65)	1.33 (1.23–1.43)
	Per 1-SD increase	1.16 (1.15–1.17)	1.16 (1.15–1.17)	1.13 (1.11–1.16)
	Monocyte count			
	Q1	Ref.	Ref.	Ref.
	Q2	1.07 (1.01–1.13)	1.04 (0.97–1.12)	1.00 (0.92–1.08)
	Q3	1.15 (1.09–1.21)	1.13 (1.06–1.21)	1.02 (0.94–1.09)
	Q4	1.52 (1.44–1.60)	1.44 (1.35–1.53)	1.17 (1.09–1.26)
	Per 1-SD increase	1.01 (1.01–1.02)	1.01 (1.01–1.02)	1.05 (1.04–1.06)
	Lymphocyte count			
	Q1	Ref.	Ref.	Ref.
	Q2	0.89 (0.85–0.94)	0.89 (0.84–0.94)	0.86 (0.80–0.92)
	Q3	0.89 (0.84–0.94)	0.88 (0.82–0.93)	0.80 (0.74–0.86)
	Q4	1.09 (1.03–1.14)	1.00 (0.94–1.07)	0.85 (0.79–0.91)
	Per 1-SD increase	1.01 (1.00–1.02)	1.00 (0.98–1.02)	0.94 (0.91–0.98)
	CRP			
	Q1	Ref.	Ref.	Ref.
	Q2	1.22 (1.14–1.3)	1.19 (1.11–1.28)	1.05 (0.97–1.14)
	Q3	1.54 (1.45–1.64)	1.46 (1.36–1.57)	1.17 (1.08–1.27)
	Q4	2.35 (2.22–2.48)	2.10 (1.96–2.24)	1.43 (1.32–1.55)
	Per 1-SD increase	1.15 (1.14–1.16)	1.14 (1.12–1.15)	1.09 (1.08–1.11)
	LMR			
	Q1	Ref.	Ref.	Ref.
	Q2	0.83 (0.78–0.87)	0.83 (0.78–0.88)	0.86 (0.80–0.92)
	Q3	0.76 (0.71–0.80)	0.74 (0.69–0.79)	0.77 (0.71–0.82)
	Q4	0.74 (0.70–0.78)	0.72 (0.67–0.77)	0.75 (0.69–0.81)
	Per 1-SD increase	0.91 (0.88–0.95)	0.91 (0.87–0.95)	0.92 (0.87–0.96)
	NLR			
	Q1	Ref.	Ref.	Ref.
	Q2	1.09 (1.03–1.15)	1.09 (1.02–1.17)	1.09 (1.01–1.18)
	Q3	1.21 (1.14–1.28)	1.17 (1.10–1.26)	1.13 (1.05–1.22)
	Q4	1.58 (1.50–1.66)	1.55 (1.46–1.65)	1.46 (1.36–1.56)
	Per 1-SD increase	1.06 (1.06–1.07)	1.08 (1.07–1.09)	1.08 (1.07–1.08)
	PLR			
	Q1	Ref.	Ref.	Ref.
	Q2	0.87 (0.82–0.91)	0.90 (0.84–0.96)	0.96 (0.89–1.03)
	Q3	0.85 (0.80–0.89)	0.89 (0.83–0.95)	1.00 (0.93–1.07)
	Q4	0.94 (0.89–0.99)	0.98 (0.91–1.03)	1.12 (1.05–1.20)
	Per 1-SD increase	1.02 (1.01–1.04)	1.03 (1.02–1.05)	1.05 (1.04–1.06)
	SII			
	Q1	Ref.	Ref.	Ref.
	Q2	1.01 (0.95–1.07)	1.03 (0.96–1.10)	1.02 (0.94–1.09)
	Q3	1.11 (1.05–1.17)	1.10 (1.03–1.17)	1.07 (0.99–1.14)
	Q4	1.40 (1.33–1.47)	1.37 (1.29–1.45)	1.28 (1.20–1.37)
	Per 1-SD increase	1.07 (1.07–1.08)	1.08 (1.07–1.09)	1.07 (1.06–1.08)
	SIRI			
	Q1	Ref.	Ref.	Ref.
	Q2	1.17 (1.10–1.25)	1.16 (1.08–1.25)	1.11 (1.02–1.21)
	Q3	1.37 (1.29–1.46)	1.32 (1.22–1.42)	1.19 (1.09–1.29)
	Q4	1.83 (1.72–1.94)	1.75 (1.63–1.87)	1.47 (1.36–1.59)
	Per 1-SD increase	1.03 (1.03–1.03)	1.03 (1.02–1.03)	1.02 (1.02–1.03)

CI, confidence intervals; CVD, cardiovascular disease; CRP, C-reactive protein; HDL, high-density lipoprotein; HR, hazard ratio; IHD, ischemic heart disease; LDL, low-density lipoprotein; LMR, Lymphocyte-to-monocyte ratio; LTCs, long-term conditions; NLR, Neutrophil-to-lymphocyte ratio; PLR, Platelet-to-lymphocyte ratio; SII, Systemic immune-inflammation index; SBP, systolic blood pressure; SD, standard deviation; SIRI, system inflammation response index; WC, waist circumference

Model 1: adjusted for age, sex, Townsend deprivation index, and ethnicity

Model 2: additionally adjusted for smoking status, weekly units of alcohol use, sleep duration, fruit and vegetable intake, processed meat intake, red meat intake, physical activity, and total sedentary time

Model 3: additionally adjusted for number of long-term conditions, HDL, total cholesterol, SBP, HbA1c, BMI, and WC

### Systemic inflammation biomarkers and ischaemic heart disease

When comparing the highest to the lowest quintiles, significantly positive associations were found for neutrophil, monocyte and lymphocyte counts, CRP, NLR, LMR, and SII, and a negative association was observed for PLR (Table 2). A non-linear relationship was observed for lymphocyte count (*P*_non−linearity_ < 0.001), CRP (*P*_non−linearity_ = 0.006), LMR (*P*_non−linearity_ < 0.001), and PLR (*P*_non−linearity_ < 0.001) (Supplementary Fig. 1), which showed both lower and higher levels were associated with an increased risk of IHD. A linear relationship was found for neutrophil count (*P*_non−linearity_ = 0.168), monocyte count (*P*_non−linearity_ = 0.914), NLR (*P*_non−linearity_ = 0.167), SII (*P*_non−linearity_ = 0.051) and SIRI (*P*_non−linearity_ = 0.054) (Supplementary Fig. 2): risk of incident IHD was 7% (HR 1.07, 95% CI 1.05–1.08), 3% (HR 1.03, 95% CI 1.02–1.04), 4% (HR 1.04, 95% CI 1.03–1.05), 3% (HR 1.03, 95% CI 1.02–1.04), and 1% (HR 1.01, 95% CI 1.01–1.02) higher per 1 SD increment of neutrophils, monocytes, NLR, SII, and SIRI respectively (Table 2).

### Systemic inflammation biomarkers and stroke

When comparing the highest to the lowest quintiles, we found significant positive associations with neutrophil count, monocyte count, CRP, and SII, a negative associations with LMR, and no significant associations with lymphocyte count, NLR, and PLR (Table 2). A non-linear relationship was observed for neutrophils (*P*_non−linearity_ = 0.001), lymphocytes (*P*_non−linearity_ < 0.001), LMR (*P*_non−linearity_ = 0.003), and PLR (*P*_non−linearity_ = 0.005) (Supplementary Fig. 3), which showed both lower and higher levels of neutrophils, lymphocytes, and PLR were associated with an increased risk of stroke. A linear relationships was found for monocytes (*P*_non−linearity_ = 0.540), NLR (*P*_non−linearity_ = 0.166), SII (*P*_non−linearity_ = 0.166), and SIRI (*P*_non−linearity_ = 0.699) (Supplementary Fig. 2): the risk of stroke increased by 4% (HR 1.04, 95% CI 1.02–1.05), 4% (HR 1.04, 95% CI 1.02–1.06), 5% (HR 1.05, 95% CI 1.03–1.06), and 2% (HR 1.02, 95% CI 1.00–1.03) per 1 SD increment in monocytes, NLR, SII and SIRI, respectively (Table 2).

### Systemic inflammation biomarkers and heart failure

Compared to the lowest quintile, higher quintiles of neutrophil count, monocyte count, CRP, NLR, PLR, and SII were associated with a higher risk of heart failure; whilst higher quintiles of lymphocyte count and LMR were associated with a lower risk (Table 2). A non-linear relationships was observed for monocyte count (*P*_non−linearity_ = 0.021), lymphocyte count (*P*_non−linearity_ < 0.001), CRP (*P*_non−linearity_ < 0.001), LMR (*P*_non−linearity_ < 0.001), and PLR (*P*_non−linearity_ < 0.001) (Supplementary Fig. 4). The relationship between lymphocyte count and heart failure showed the increased risk in lower levels but decreased risk in the middle range. A linear relationships was found for neutrophil count (*P*_non−linearity_ = 0.132), NLR (*P*_non−linearity_ = 0.377), and SII (*P*_non−linearity_ = 0.166) (Supplementary Fig. 2).

### Predictive performance of inflammatory markers

Table 3 shows the predictive performance of different inflammatory biomarkers. Our results indicated that all the derived biomarkers (NLR, LMR, PLR, and SII) except for SIRI had higher C-statistics than neutrophil count for CVD, IHD, and heart failure (*P*_comparison_ < 0.05), and NLR, PLR, and SII also had higher C-statistics for stroke. SII had the highest C-statistic for CVD and IHD, PLR has the highest C-statistic for stroke, while lymphocyte count had the largest C-statistic for heart failure. Compared to a model of CVD prediction with classical risk factors (C-index = 0.702), discrimination was improved on the addition of inflammation markers for CVD (C-index change 0.0069, 95% CI 0.0033 to 0.0107), IHD (C-index change 0.0054, 95% CI 0.0013 to 0.0095), and heart failure (C-index change 0.0153, 95% CI 0.0089 to 0.0218) (Table 4 and Supplemental Table 5).

**Table 3:** Predictive performance of systemic inflammation biomarkers for incident CVD

Outcome	Exposure	C-statistic (95% CI)	*P* for comparison
CVD	Neutrophil count	0.439 (0.434–0.445)	Ref.
	Monocyte count	0.428 (0.422–0.433)	< 0.001
	Lymphocyte count	0.485 (0.479–0.491)	< 0.001
	CRP	0.418 (0.413–0.424)	< 0.001
	LMR	0.448 (0.442–0.454)	0.028
	NLR	0.464 (0.458–0.470)	< 0.001
	PLR	0.465 (0.459–0.471)	< 0.001
	SII	0.487 (0.481–0.493)	< 0.001
	SIRI	0.427 (0.421–0.433)	< 0.001
IHD	Neutrophil count	0.444 (0.438–0.450)	Ref.
	Monocyte count	0.432 (0.426–0.438)	< 0.001
	Lymphocyte count	0.480 (0.474–0.487)	< 0.001
	CRP	0.421 (0.415–0.427)	< 0.001
	LMR	0.456 (0.449–0.462)	< 0.001
	NLR	0.471 (0.465–0.478)	< 0.001
	PLR	0.466 (0.460–0.473)	< 0.001
	SII	0.488 (0.482–0.495)	< 0.001
	SIRI	0.435 (0.428–0.441)	< 0.001
Stroke	Neutrophil count	0.458 (0.447–0.470)	Ref.
	Monocyte count	0.440 (0.429–0.452)	< 0.001
	Lymphocyte count	0.496 (0.483–0.508)	< 0.001
	CRP	0.439 (0.427–0.450)	< 0.001
	LMR	0.451 (0.439–0.463)	0.013
	NLR	0.471 (0.459–0.483)	< 0.001
	PLR	0.515 (0.503–0.527)	< 0.001
	SII	0.483 (0.471–0.495)	< 0.001
	SIRI	0.440 (0.429–0.452)	< 0.001
Heart failure	Neutrophil count	0.415 (0.405–0.425)	Ref.
	Monocyte count	0.417 (0.406–0.427)	0.954
	Lymphocyte count	0.505 (0.494–0.516)	< 0.001
	CRP	0.382 (0.372–0.392)	< 0.001
	LMR	0.422 (0.412–0.433)	0.196
	NLR	0.429 (0.419–0.440)	< 0.001
	PLR	0.481 (0.470–0.492)	< 0.001
	SII	0.459 (0.448–0.470)	< 0.001
	SIRI	0.395 (0.384–0.406)	< 0.001

CRP, C-reactive protein; IHD, ischemic heart disease; LMR, Lymphocyte-to-monocyte ratio; NLR, Neutrophil-to-lymphocyte ratio; SII, Systemic immune-inflammation index; PLR, Platelet-to-lymphocyte ratio

**Table 4:** Change in C-index for prediction of incident CVD on addition of inflammation biomarkers

Exposure	C-index	C-index change (95% CI)
Traditional risk factors model	0.702	–
+ Single inflammation biomarkers		
Neutrophil count	0.705	0.0025 (−0.0022 to 0.0062)
Monocyte count	0.703	0.0015 (−0.0022 to 0.0052)
Lymphocyte count	0.703	0.0002 (−0.0035 to 0.0039)
CRP	0.706	0.0043 (0.0006 to 0.0079)
LMR	0.703	0.0005 (−0.0032 to 0.0042)
NLR	0.703	0.0008 (−0.0029 to 0.0045)
PLR	0.703	0.0006 (−0.0031 to 0.0043)
SII	0.703	0.0004 (−0.003 to 0.0042)
SIRI	0.704	0.0018 (−0.0018 to 0.0056)
+ traditional inflammation biomarkers (Neutrophil count, monocyte count, lymphocyte count, CRP)	0.708	0.0060 (0.0023 to 0.0097)
+ all the inflammation biomarkers (Neutrophil count, monocyte count, lymphocyte count, CRP, LMR, NLR, PLR, SII, SIRI)	0.709	0.0069 (0.0033 to 0.0107)

CRP, C-reactive protein; LMR, Lymphocyte-to-monocyte ratio; NLR, Neutrophil-to-lymphocyte ratio; SII, Systemic immune-inflammation index; SIRI, system inflammation response index; PLR, Platelet-to-lymphocyte ratio

### Sensitivity analyses

The sensitivity analyses excluding participants with incident CVD in the first two years produced similar results (Supplemental Table 6). The sensitivity analyses with further adjustment for insulin, and antihypertensive and cholesterol-lowering medications, showed similar results, except that the association between SII and lymphocyte count and CVD, the association between lymphocyte count and PLR and IHD became non-significant. When performing the analysis by excluding participants who had statin therapy at baseline, the results were consistent, except that the association between neutrophil count and stroke became non-significant. When using the imputing data of the missing covariates at baseline or adjusting for Downtown deprivation index as continuous variable instead of categorical variable, the model also showed the stable findings (Supplemental Table 5).

## Discussion

In this large, prospective population cohort study we corroborated known associations between well-established biomarkers– neutrophil count, monocyte count, lymphocyte count, and CRP– and CVD and CVD sub-types but also demonstrated positive associations for the novel biomarkers of NLR, PLR, SII, and SIRI and negative associations for LMR. NLR, PLR, SII, SIRI, and LMR were associated with all four of the outcomes investigated. Relationships were linear for two of the existing biomarkers (neutrophil and monocyte counts). However, non-linear relationships were observed for CRP, lymphocyte count, NLR, LMR, PLR, SII, and SIRI. Compared with the more established biomarkers, all the novel biomarkers except for SIRI had statistically superior predictive performance for three of the outcomes investigated. Addition of these inflammation biomarkers together showed improved discrimination of the traditional risk prediction model.

Inflammation plays a significant role in the pathogenesis of CVD [23] and immune cells such as neutrophils, lymphocytes, monocytes, and macrophages mediate local responses to tissue damage and infection and are an important driver of CVD [24, 25]. Many observational studies have explored associations between white blood cell counts (e.g., neutrophils and leukocytes) and CVD [26–30], but the associations between novel systematic inflammatory biomarkers and risk of CVD have been poorly investigated [31–33]. A cross-sectional study including 280 asymptomatic participants of a cardiovascular screening programme found an independent association between NLR and subclinical coronary disease among obese individuals [31]. In around 40,000 eligible participants from the National Health and Nutrition Examination Survey, SII was found to be associated with cardiovascular mortality [32], and LMR and PLR were inversely associated with risk of coronary heart disease [33]. The findings were consistent with those in the present study. Our study expanded the existing evidence by investigating a series of systematic inflammatory biomarkers and a range of CVD outcomes, and exploring the nature of the relationship in terms of direction, strength, linearity and dose–response relationship. Moreover, previous studies investigating the systematic inflammatory biomarkers and CVD mortality and all-cause mortality had limited sample size and have not explored whether systematic inflammatory biomarkers can enhance the discrimination of risk for the traditional risk factors prediction model [32]. This suggest that future studies are warranted to explore the prediction value of inflammation biomarkers for all-cause mortality and CVD specific mortality.

In this study, we investigated the dose–response relationship between inflammation markers and the risk of CVD, revealing a significant nonlinear (e.g., U-shaped) relationship between most inflammation biomarkers and CVD risk. The findings have important implications for clinical practice and public health, underscoring the importance of considering the full spectrum of inflammation in cardiovascular health and highlighting the necessity for healthcare providers to monitor both low and high levels of inflammation markers (e.g., lymphocyte count, PLR, SII), as both can negatively impact cardiovascular health.

This study is also the first to evaluate the predictive performance of these inflammatory biomarkers for incident CVD. We found that the novel derived biomarkers (NLR, LMR, PLR, and SII) had better ability to predict CVD and CVD subtypes compared to neutrophil count. A potential explanation for these findings could be that NLR, LMR, PLR, and SII indicate the balance between innate immunity and adaptive immunity but neutrophil count only represents the innate immunity [34, 35]. Therefore, our study suggests that the derived biomarkers provide valuable insights into the immune response’s balance in terms of inflammation and adaptive immunity and could serve as potential biomarkers for prevention or early detection of CVD.

This study investigated the association between inflammation biomarkers and CVD, highlighting their potential role in risk assessment. While our findings provided preliminary evidence on the significant association of different inflammation biomarkers in CVD and its subtypes, future studies are needed to evaluate their influence on statin therapy decisions or the need for coronary calcium scoring. By comparing their predictive performance using the C-index, we assessed differences among these biomarkers and provided evidence of improved discrimination of the traditional risk prediction model with addition of a comprehensive of inflammation biomarkers, offering insights for future research on their integration into established risk models such as Systematic Coronary Risk Evaluation 2 (SCORE2) and the Prediction for Atherosclerotic Cardiovascular Disease Risk Events (PREVENT) score. Future studies should focus on refining risk prediction models, assessing their incremental predictive value beyond traditional risk factors; however, caution is needed when interpreting predictive value due to the nonlinear association.

This study has several strengths. Firstly, this study is a prospective cohort study, with a large sample size and long follow-up period. Second, we were able to investigate a range of systemic inflammation biomarkers, including CRP and differential leukocyte count, and composite measures such as LMR, NLR, PLR, and SII, as well as a range of outcomes: incident CVD and its main subtypes, including IHD, stroke and heart failure. Third, our study explored dose–relationships, adjusted for demographic, lifestyle and health confounders, performed a series of sensitivity analyses, and compared the predictive performance across biomarkers.

However, there were some limitations to be noted. First, caution should be taken when generalizing these findings to other populations because UK Biobank participants were adults aged 40–69 years and primarily of White British origin. There was an also healthy volunteer bias that should be considered when using UK Biobank, although effect-size estimates should be generalizable as validated in previous studies [36]. Second, the inflammatory measures could have changed over time but they were only assessed at the baseline. Previous studies have identified significant positive associations between changes in neutrophil and monocyte counts and the risk of CVD [37]. Future studies should explore whether dynamic changes in systematic inflammation impact the risk of CVD. Third, measurement of confounders such as diet and physical activity were based on self-reported data, which may lead to inaccuracies but is unlikely to lead to systematic bias in relation to serum biomarker measurements. Finally, residual confounding cannot be ruled out given the nature of observational studies, even though we have adjusted for a range of confounding factors.

## Conclusions

Novel systemic inflammation biomarkers were significantly associated with CVD, IHD, stroke and heart failure independent of confounders, with evidence of dose-relationships. Further studies are needed to explore the potential of the inclusion of systemic inflammation biomarkers when constructing predictive models of CVD and for monitoring biomarkers of systemic inflammation as part of the prevention and early management of CVD.

## Supplementary Information

Below is the link to the electronic supplementary material.

Supplementary Material 1

## Data Availability

Data analysed in this study was from the UK Biobank and is available upon application to UK Biobank https://www.ukbiobank.ac.uk.

## Abbreviations

BMI: Body mass index
CI: Confidence interval
CRP: C-reactive protein
CVD: Cardiovascular disease
DBP: Diastolic blood pressure
HDL: High-density lipoprotein cholesterol
HR: Hazard ratio
HDL: High-density lipoprotein cholesterol
IHD: Ischemic heart disease
LDL: Low-density lipoprotein
LMR: Lymphocyte-to monocyte ratio
NLR: Neutrophil-to-lymphocyte ratio
PLR: Platelet-to-lymphocyte ratio
SBP: Systolic blood pressure
SII: Systemic immune inflammation index
SIRI: System inflammation response index
WC: Waist circumference
