# Supplementary material for: Association between systemic inflammation biomarkers and incident cardiovascular disease in 423,701 individuals: evidence from the UK biobank cohort
Source: Cardiovasc Diabetol. 2025 Apr 15;24:162. doi: 10.1186/s12933-025-02721-9 (PMC12001404; doi:10.1186/s12933-025-02721-9)
Supplement: Supplementary file 1 — Supplementary Material 1 [file 12933_2025_2721_MOESM1_ESM.docx]

**Supplementary Material**

**Association between systemic inflammation biomarkers and incident cardiovascular disease in 423,701 individuals: evidence from the UK Biobank cohort**

Pei QIN^1^, Frederick K Ho^1^, Carlos Celis-Morales^2,3,4^, Jill P Pell^1*^

1. School of Health and Wellbeing, University of Glasgow, Glasgow, UK.

2. School of Cardiovascular and Metabolic Health, University of Glasgow, Glasgow UK.

3. Human Performance Lab, Education, Physical Activity and Health Research Unit, University Católica del Maule, Talca, Chile.

4. Centro de Investigación en Medicina de Altura (CEIMA), Universidad Arturo Prat, Iquique, Chile.

**Corresponding Author：**

Professor Jill Pell CBE

School of Health and Wellbeing, Clarice Pears Building, 90 Byres Road, University of Glasgow, G12 8TB, United Kingdom

Email: Jill.Pell@glasgow.ac.uk

**Supplementary Table 1. List of self-reported long-term conditions considered for multimorbidity count**

| **Long term condition grouping** | **Conditions included as reported by participants** |
| --- | --- |
| 1. Painful conditions | Back pain  Joint pain  Back pain  Joint pain  Headaches (not migraine)  Sciatica  Plantar fasciitis  Carpal tunnel syndrome  Fibromyalgia  Arthritis  Shingles  Disc problem  Prolapsed disc/slipped disc  Spine arthritis/spondylitis  Ankylosing spondylitis  Back problem  Osteoarthritis  Gout  Cervical spondylosis  Trigeminal neuralgia  Disc degeneration  Trapped nerve/compressed nerve |
| 1. Hypertension | Hypertension  Essential Hypertension |
| 1. Depression | Depression  Postnatal Depression |
| 1. Asthma | Asthma |
| 1. Atrial Fibrillation | Atrial Fibrillation |
| 1. Coronary Heart Disease | Heart attack/Myocardial Infarction  Angina |
| 1. Dyspepsia | Gastro-oesophageal reflux (GORD)/gastric reflux  Oesophagitis /Barrett's oesophagus  Gastric stomach ulcers  Gastric erosions/gastritis  Duodenal ulcer  Dyspepsia/indigestion  Hiatus hernia  Helicobacter pylori |
| 1. Diabetes | Diabetic nephropathy  Diabetic neuropathy/ulcers  Diabetes  Type 1 diabetes  Type 2 diabetes  Diabetic eye disease |
| \| 1. Thyroid disorders \| \| --- \| | Thyroid problem (not cancer)  Hyperthyroidism/thyrotoxicosis  Hypothyroidism/myxoedema  Grave’s disease  Thyroid goitre  Thyroiditis |
| 1. Connective tissue disorders | Myositis/myopathy  Systemic Lupus Erythematosus  Connective tissue disorder  Sjogrens syndrome/sicca syndrome  Dermatopolymyositis  Scleroderma/systemic sclerosis  Rheumatoid arthritis  Psoriatic arthropathy  Dermatomyositis  Polymyositis  Polymyalgia Rheumatica  Malabsorption/coeliac disease |
| 1. Chronic Obstructive Pulmonary Disease (COPD) | COPD/chronic obstructive airways disease  Emphysema/chronic bronchitis  Emphysema |
| 1. Anxiety | Anxiety/panic attacks  Nervous breakdown  Post-traumatic stress disorder  Obsessive compulsive disorder  Stress  Insomnia  Psychological/psychiatric problem |
| 1. Irritable bowel syndrome | Irritable bowel syndrome |
| 1. Alcohol problems | Alcohol dependency  Alcoholic liver disease/alcoholic cirrhosis |
| 1. Other psychoactive substance abuse | Opioid dependency  Other substance abuse/dependency |
| 1. Treated constipation | Constipation |
| 1. Stroke/Transient Ischaemic Attack (TIA) | Stroke  TIA  Subarachnoid haemorrhage  Brain haemorrhage  Ischaemic stroke |
| 1. Chronic kidney disease | Polycystic kidney  Diabetic nephropathy  Renal/kidney failure  Renal failure requiring dialysis  Renal failure not requiring dialysis  Kidney nephropathy  Immunoglobulin A (IgA) nephropathy |
| 1. Diverticular disease | Diverticular disease  Diverticulitis |
| 1. Peripheral vascular disease | Peripheral vascular disease  Leg claudication/intermittent claudication |
| 1. Heart failure | Cardiomyopathy  Hypertrophic cardiomyopathy  Heart failure/pulmonary oedema |
| 1. Prostate disorders | Prostate problem (not cancer)  Enlarged prostate  Benign prostatic hypertrophy |
| 1. Glaucoma | Glaucoma |
| 1. Epilepsy | Epilepsy |
| 1. Dementia | Dementia  Alzheimer’s disease  Cognitive impairment |
| 1. Schizophrenia/bipolar disorder | Schizophrenia  Mania/  Bipolar disorder  Manic depression |
| 1. Psoriasis/eczema | Eczema  Dermatitis  Psoriasis |
| 1. Inflammatory Bowel Disease | Inflammatory Bowel Disease  Crohn’s disease  Ulcerative colitis |
| 1. Migraine | Migraine |
| 1. Chronic sinusitis | Chronic sinusitis |
| 1. Anorexia or bulimia | Anorexia  Bulimia  Other eating disorders |
| 1. Bronchiectasis | Bronchiectasis |
| 1. Parkinson’s disease | Parkinson’s disease |
| 1. Multiple Sclerosis | Multiple Sclerosis |
| 1. Viral Hepatitis | Infective/viral hepatitis  Hepatitis B  Hepatitis C  Hepatitis D  Hepatitis E |
| 1. Chronic Liver disease | Oesophageal varices  Non infective hepatitis  Liver failure/cirrhosis  Primary biliary cirrhosis |
| 1. Osteoporosis | Osteoporosis |
| 1. Chronic fatigue syndrome | Chronic fatigue syndrome |
| 1. Endometriosis | Endometriosis |
| 1. Meniere’s disease | Meniere’s disease |
| 1. Pernicious Anaemia | Pernicious Anaemia |
| 1. Polycystic ovary | Polycystic ovary |
| 1. Cancer | Lifetime diagnosis |

**Supplementary Table 2.** **Baseline characteristics of participants by CVD subtypes in the UK Biobank**

|  | **IHD** | | **p** | **Stroke** | | **p** | **Heart failure** | | **p** |
| --- | --- | --- | --- | --- | --- | --- | --- | --- | --- |
|  | **No** | **Yes** |  | **No** | **Yes** |  | **No** | **Yes** |  |
| N | 391105 | 32596 |  | 414024 | 9677 |  | 411458 | 12243 |  |
| Age (years), mean (SD) | 55.86 (8.10) | 59.88 (7.00) | <0.001 | 56.05 (8.08) | 60.93 (6.82) | <0.001 | 56.01 (8.08) | 61.54 (6.46) | <0.001 |
| Men, n (%) | 167928 (42.9) | 20095 (61.6) | <0.001 | 182624 (44.1) | 5399 (55.8) | <0.001 | 180861 (44.0) | 7162 (58.5) | <0.001 |
| Deprivation index, n (%) |  |  | <0.001 |  |  | <0.001 |  |  | <0.001 |
| Low | 133596 (34.2) | 10239 (31.4) |  | 140765 (34.0) | 3070 (31.8) |  | 140259 (34.1) | 3576 (29.2) |  |
| Moderate | 131189 (33.6) | 10827 (33.3) |  | 138821 (33.6) | 3195 (33.1) |  | 138006 (33.6) | 4010 (32.8) |  |
| High | 125824 (32.2) | 11493 (35.3) |  | 133917 (32.4) | 3400 (35.2) |  | 132670 (32.3) | 4647 (38.0) |  |
| Ethnicity (%) |  |  | <0.001 |  |  | 0.133 |  |  | 0.32 |
| White | 369083 (96.9) | 30661 (96.5) |  | 390527 (96.9) | 9217 (97.2) |  | 388132 (96.9) | 11612 (96.8) |  |
| South Asia | 5572 (1.5) | 789 (2.5) |  | 6232 (1.5) | 129 (1.4) |  | 6158 (1.5) | 203 (1.7) |  |
| Other | 6177 (1.6) | 328 (1.0) |  | 6372 (1.6) | 133 (1.4) |  | 6325 (1.6) | 180 (1.5) |  |
| Smoking status, n (%) |  |  | <0.001 |  |  | <0.001 |  |  | <0.001 |
| Never | 220319 (56.6) | 15048 (46.5) |  | 230799 (56.0) | 4568 (47.5) |  | 230055 (56.2) | 5312 (43.7) |  |
| Previous | 129815 (33.3) | 12834 (39.6) |  | 139027 (33.7) | 3622 (37.7) |  | 137632 (33.6) | 5017 (41.20) |  |
| Current | 39156 (10.1) | 4512 (13.9) |  | 42245 (10.3) | 1423 (14.8) |  | 41831 (10.2) | 1837 (15.1) |  |
| Alcohol (weekly units), median (IQR) | 10.50 (3.00, 22.20) | 11.40 (1.50, 25.20) | 0.001 | 10.50 (3.00, 22.50) | 11.70 (1.50, 25.80) | <0.001 | 10.50 (3.00, 22.50) | 10.80 (1.50, 25.80) | <0.001 |
| Physical activity, n (%) |  |  | <0.001 |  |  | 0.003 |  |  | <0.001 |
| Low | 58011 (18.3) | 5316 (20.6) |  | 61853 (18.4) | 1474 (19.3) |  | 61224 (18.3) | 2103 (22.3) |  |
| Moderate | 129999 (40.9) | 10180 (39.4) |  | 137198 (40.9) | 2981 (39.0) |  | 136417 (40.9) | 3762 (39.9) |  |
| High | 129506 (40.8) | 10350 (40.0) |  | 136661 (40.7) | 3195 (41.8) |  | 136299 (40.8) | 3557 (37.8) |  |
| Sedentary time (hours), mean (SD) | 4.43 (2.56) | 4.94 (2.70) | <0.001 | 4.46 (2.57) | 4.77 (2.65) | <0.001 | 4.45 (2.57) | 4.98 (2.72) | <0.001 |
| Fruit and vegetable intake (portion per day), mean (SD) | 4.12 (2.43) | 4.01 (2.52) | <0.001 | 4.11 (2.43) | 4.06 (2.55) | 0.028 | 4.11 (2.43) | 4.09 (2.61) | 0.242 |
| Processed meat intake (times/week), n (%) |  |  | <0.001 |  |  | <0.001 |  |  | <0.001 |
| 0 | 37391 (9.6) | 2374 (7.3) |  | 38995 (9.5) | 770 (8.0) |  | 38941 (9.5) | 824 (6.8) |  |
| 0-2 | 233930 (60.0) | 18589 (57.3) |  | 246960 (59.9) | 5559 (57.6) |  | 245685 (59.9) | 6834 (56.1) |  |
| >2 | 118460 (30.4) | 11483 (35.4) |  | 126629 (30.7) | 3314 (34.4) |  | 125416 (30.6) | 4527 (37.2) |  |
| Red meat intake (times/week), n (%) |  |  | <0.001 |  |  | <0.001 |  |  | <0.001 |
| 0 | 27388 (7.1) | 1582 (4.9) |  | 28486 (7.0) | 484 (5.1) |  | 28415 (7.0) | 555 (4.6) |  |
| 0-2 | 237899 (61.6) | 19029 (59.4) |  | 251288 (61.5) | 5640 (59.2) |  | 249947 (61.5) | 6981 (58.1) |  |
| >2 | 121108 (31.30) | 11436 (35.7) |  | 129144 (31.6) | 3400 (35.7) |  | 128057 (31.5) | 4487 (37.3) |  |
| Sleep duration (hours/day), n (%) |  |  | <0.001 |  |  | <0.001 |  |  | <0.001 |
| 1-6 | 93756 (24.1) | 8895 (27.6) |  | 100179 (24.4) | 2472 (25.8) |  | 99302 (24.3) | 3349 (27.7) |  |
| 7-8 | 267049 (68.8) | 20449 (63.3) |  | 281293 (68.4) | 6205 (64.7) |  | 280017 (68.5) | 7481 (61.8) |  |
| ≥9 | 27608 (7.1) | 2938 (9.1) |  | 29635 (7.2) | 911 (9.5) |  | 29264 (7.2) | 1282 (10.6) |  |
| BMI (kg/m2), mean (SD) | 27.17 (4.69) | 28.58 (4.91) | <0.001 | 27.26 (4.72) | 27.94 (4.88) | <0.001 | 27.21 (4.67) | 29.51 (5.80) | <0.001 |
| WC (cm), mean (SD) | 89.28 (13.17) | 95.33 (13.32) | <0.001 | 89.66 (13.26) | 93.31 (13.49) | <0.001 | 89.50 (13.16) | 97.70 (14.81) | <0.001 |
| HDL (mmol/L), mean (SD) | 1.47 (0.38) | 1.34 (0.35) | <0.001 | 1.46 (0.38) | 1.41 (0.38) | <0.001 | 1.46 (0.38) | 1.37 (0.38) | <0.001 |
| TG (mmol/L), mean (SD) | 1.71 (1.00) | 2.01 (1.12) | <0.001 | 1.73 (1.01) | 1.85 (1.03) | <0.001 | 1.73 (1.01) | 1.92 (1.09) | <0.001 |
| LDL (mmol/L), mean (SD) | 3.61 (0.84) | 3.64 (0.92) | <0.001 | 3.61 (0.85) | 3.58 (0.88) | 0.006 | 3.61 (0.85) | 3.46 (0.91) | <0.001 |
| Total Cholesterol (mmol/L), mean (SD) | 5.77 (1.10) | 5.74 (1.21) | <0.001 | 5.76 (1.11) | 5.70 (1.16) | <0.001 | 5.77 (1.11) | 5.54 (1.20) | <0.001 |
| HbA1c (mmol/mol), mean (SD) | 35.66 (6.06) | 37.99 (8.81) | <0.001 | 35.80 (6.28) | 37.67 (8.47) | <0.001 | 35.75 (6.18) | 38.87 (10.01) | <0.001 |
| SBP (mmHg), mean (SD) | 137.15 (18.48) | 144.04 (18.57) | <0.001 | 137.50 (18.51) | 145.54 (19.68) | <0.001 | 137.46 (18.50) | 145.28 (19.45) | <0.001 |
| DBP (mmHg), mean (SD) | 82.20 (10.06) | 84.17 (10.20) | <0.001 | 82.31 (10.06) | 84.54 (10.67) | <0.001 | 82.31 (10.06) | 83.82 (10.58) | <0.001 |
| Number of long-term conditions, n (%) |  |  | <0.001 |  |  | <0.001 |  |  | <0.001 |
| 0 | 148731 (38.0) | 7325 (22.5) |  | 153695 (37.1) | 2361 (24.4) |  | 153826 (37.4) | 2230 (18.2) |  |
| 1 | 132989 (34.0) | 10510 (32.2) |  | 140275 (33.9) | 3224 (33.3) |  | 139875 (34.0) | 3624 (29.6) |  |
| ≥2 | 109385 (28.0) | 14761 (45.3) |  | 120054 (29.0) | 4092 (42.3) |  | 117757 (28.6) | 6389 (52.2) |  |
| Diabetes, n (%) | 14609 (3.7) | 3234 (9.9) | <0.001 | 16965 (4.1) | 878 (9.1) | <0.001 | 16270 (4.0) | 1573 (12.8) | <0.001 |
| Hypertension, n (%) | 90051 (23.0) | 13319 (40.9) | <0.001 | 99572 (24.0) | 3798 (39.2) | <0.001 | 97693 (23.7) | 5677 (46.4) | <0.001 |
| Hyperlipidemia, n (%) | 38194 (9.8) | 6047 (18.6) | <0.001 | 42729 (10.3) | 1512 (15.6) | <0.001 | 41971 (10.2) | 2270 (18.5) | <0.001 |
| Neutrophil count (10^9 cells/L), mean (SD) | 4.17 (1.39) | 4.45 (1.48) | <0.001 | 4.19 (1.40) | 4.40 (1.52) | <0.001 | 4.18 (1.39) | 4.62 (1.61) | <0.001 |
| Monocyte count (10^9 cells/L), mean (SD) | 0.47 (0.28) | 0.51 (0.24) | <0.001 | 0.47 (0.28) | 0.51 (0.23) | <0.001 | 0.47 (0.27) | 0.52 (0.38) | <0.001 |
| Lymphocyte count (10^9 cells/L), mean (SD) | 1.96 (1.13) | 2.02 (1.46) | <0.001 | 1.96 (1.15) | 1.99 (1.47) | 0.009 | 1.96 (1.16) | 1.97 (0.97) | 0.439 |
| NLR, mean (SD) | 2.33 (1.18) | 2.46 (1.31) | <0.001 | 2.34 (1.18) | 2.48 (1.39) | <0.001 | 2.33 (1.17) | 2.65 (1.61) | <0.001 |
| LMR, mean (SD) | 4.67 (3.79) | 4.46 (7.90) | <0.001 | 4.66 (4.27) | 4.37 (3.18) | <0.001 | 4.67 (4.25) | 4.28 (4.24) | <0.001 |
| PLR, mean (SD) | 142.79 (60.24) | 138.56 (60.18) | <0.001 | 142.44 (60.08) | 143.16 (66.81) | 0.245 | 142.44 (59.78) | 143.01 (74.14) | 0.308 |
| SII, mean (SD) | 594.82 (349.91) | 619.00 (401.77) | <0.001 | 595.86 (352.38) | 631.74 (424.33) | <0.001 | 594.58 (349.50) | 667.09 (481.85) | <0.001 |
| SIRI, median (IQR) | 0.93 (0.66, 1.32) | 1.06 (0.74, 1.50) | <0.001 | 0.94 (0.67, 1.33) | 1.05 (0.73, 1.50) | <0.001 | 0.94 (0.66, 1.32) | 1.14 (0.80, 1.65) | <0.001 |
| CRP (mg/L), median (IQR) | 1.28 (0.63, 2.65) | 1.75 (0.89, 3.51) | <0.001 | 1.3 (0.64, 2.70) | 1.63 (0.82, 3.36) | <0.001 | 1.29 (0.64, 2.68) | 2.06 (1.01, 4.30) | <0.001 |

Abbreviations: Ref, reference group; BMI, body mass index; CI, confidence interval; CRP, C-reactive protein; CVD, cardiovascular disease; DBP, diastolic blood pressure; HbA1c, glycated haemoglobin; HDL, high-density lipoprotein cholesterol; IQR, interquartile range; LDL, low-density lipoprotein; LMR, Lymphocyte-to monocyte ratio; NLR, Neutrophil-to-lymphocyte ratio; PLR, Platelet-to-lymphocyte ratio; SBP, systolic blood pressure; SII, systemic immune inflammation index; SD, standard deviation; SIRI, system inflammation response index; WC, waist circumference.

**Supplementary Table 3.** **Baseline characteristics between participants with and without missing inflammation biomarker data**

|  | **Complete-case sample** | **Missing data** | **P for difference** |
| --- | --- | --- | --- |
| N | 46927 | 455412 |  |
| Age (years), mean (SD) | 56.46 (8.14) | 56.54 (8.09) | 0.062 |
| Men, n (%) | 20204 (43.1) | 208853 (45.9) | <0.001 |
| Deprivation index, n (%) |  |  | <0.001 |
| Low | 14738 (31.4) | 152638 (33.6) |  |
| Moderate | 15290 (32.6) | 151860 (33.4) |  |
| High | 16846 (35.9) | 150344 (33.1) |  |
| Ethnicity (%) |  |  | <0.001 |
| White | 42988 (95.4) | 429558 (96.8) |  |
| South Asia | 948 (2.1) | 7116 (1.6) |  |
| Other | 1137 (2.5) | 6921 (1.6) |  |
| Smoking status, n (%) |  |  | <0.001 |
| Never | 25535 (55.2) | 247900 (54.7) |  |
| Previous | 15493 (33.5) | 157505 (34.8) |  |
| Current | 5224 (11.30) | 47734 (10.5) |  |
| Alcohol (weekly units), median (IQR) | 10.5 (2.41, 22.50) | 9.6 (1.50, 21.00) | <0.001 |
| Physical activity, n (%) |  |  | <0.001 |
| Low | 7010 (20.5) | 69178 (18.8) |  |
| Moderate | 13987 (40.9) | 149994 (40.8) |  |
| High | 13211 (38.6) | 148873 (40.4) |  |
| Sedentary time (hours), mean (SD) | 4.45 (2.68) | 4.52 (2.59) | <0.001 |
| Fruit and vegetable intake (portion per day), mean (SD) | 4.09 (2.56) | 4.11 (2.45) | 0.104 |
| Processed meat intake (times/week), n (%) |  |  | <0.001 |
| 0 | 4540 (9.8) | 42234 (9.3) |  |
| 0-2 | 27796 (60.0) | 270480 (59.6) |  |
| >2 | 13997 (30.2) | 141063 (31.10) |  |
| Red meat intake (times/week), n (%) |  |  | 0.124 |
| 0 | 3215 (7.0) | 30506 (6.8) |  |
| 0-2 | 27922 (60.9) | 275418 (61.30) |  |
| >2 | 14693 (32.1) | 143587 (31.9) |  |
| Sleep duration (hours/day), n (%) |  |  | <0.001 |
| 1-6 | 11620 (25.2) | 111585 (24.7) |  |
| 7-8 | 30691 (66.5) | 305891 (67.7) |  |
| ≥9 | 3819 (8.3) | 34519 (7.6) |  |
| BMI (kg/m2), mean (SD) | 27.64 (5.09) | 27.41 (4.77) | <0.001 |
| WC (cm), mean (SD) | 90.66 (13.91) | 90.28 (13.44) | <0.001 |
| HDL (mmol/L), mean (SD) | 1.45 (0.39) | 1.45 (0.38) | 0.28 |
| TG (mmol/L), mean (SD) | 1.79 (1.26) | 1.75 (1.02) | <0.001 |
| LDL (mmol/L), mean (SD) | 3.55 (0.88) | 3.56 (0.87) | 0.344 |
| Total Cholesterol (mmol/L), mean (SD) | 5.72 (1.18) | 5.69 (1.14) | 0.014 |
| HbA1c (mmol/mol), mean (SD) | 36.43 (7.35) | 36.11 (6.73) | <0.001 |
| SBP (mmHg), mean (SD) | 137.99 (18.98) | 137.76 (18.59) | 0.019 |
| DBP (mmHg), mean (SD) | 82.24 (10.21) | 82.19 (10.11) | 0.437 |
| Number of long-term conditions, n (%) |  |  | <0.001 |
| 0 | 16745 (35.7) | 156665 (34.4) |  |
| 1 | 15041 (32.1) | 149191 (32.8) |  |
| ≥2 | 15141 (32.3) | 149556 (32.8) |  |
| Diabetes, n (%) | 2574 (5.5) | 22918 (5.0) | <0.001 |
| Hypertension, n (%) | 12448 (26.5) | 120812 (26.5) | 0.998 |
| Hyperlipidemia, n (%) | 5403 (11.5) | 56213 (12.3) | <0.001 |
| Neutrophil count (10^9 cells/L), mean (SD) | 4.31 (1.55) | 4.22 (1.41) | <0.001 |
| Monocyte count (10^9 cells/L), mean (SD) | 0.48 (0.24) | 0.48 (0.27) | 0.444 |
| Lymphocyte count (10^9 cells/L), mean (SD) | 1.97 (1.10) | 1.97 (1.18) | 0.833 |
| NLR, mean (SD) | 2.42 (1.81) | 2.36 (1.25) | <0.001 |
| LMR, mean (SD) | 4.67 (4.15) | 4.62 (4.24) | 0.126 |
| PLR, mean (SD) | 142.15 (159.01) | 141.98 (62.12) | 0.732 |
| SII, mean (SD) | 614.22 (579.58) | 599.10 (365.75) | <0.001 |
| SIRI, median (IQR) | 0.95 (0.67, 1.35) | 0.96 (0.67, 1.37) | <0.001 |
| CRP (mg/L), median (IQR) | 1.33 (0.66, 2.76) | 1.37 (0.67, 2.87) | <0.001 |

Abbreviations: Ref, reference group; BMI, body mass index; CI, confidence interval; CRP, C-reactive protein; CVD, cardiovascular disease; DBP, diastolic blood pressure; HbA1c, glycated haemoglobin; HDL, high-density lipoprotein cholesterol; IQR, interquartile range; LDL, low-density lipoprotein; LMR, Lymphocyte-to monocyte ratio; NLR, Neutrophil-to-lymphocyte ratio; PLR, Platelet-to-lymphocyte ratio; SBP, systolic blood pressure; SII, systemic immune inflammation index; SD, standard deviation; SIRI, system inflammation response index; WC, waist circumference.

**Supplementary Table 4. Association of systemic inflammation indicators and incident CVD with further adjustment for the other systemic inflammation indicators**

| **Exposure** |  | **HR (95% CI)** |
| --- | --- | --- |
| Neutrophil count |  |  |
| Q1 |  | ref |
| Q2 |  | 1.02 (0.98-1.06) |
| Q3 |  | 1.03 (0.98-1.07) |
| Q4 |  | 1.05 (1.00-1.10) |
| Monocyte count |  |  |
| Q1 |  | ref |
| Q2 |  | 1.00 (0.96-1.04) |
| Q3 |  | 1.07 (1.03-1.11) |
| Q4 |  | 1.12 (1.07-1.17) |
| Lymphocyte count |  |  |
| Q1 |  | ref |
| Q2 |  | 1.02 (0.98-1.06) |
| Q3 |  | 0.99 (0.94-1.03) |
| Q4 |  | 1.06 (1.01-1.12) |
| CRP |  |  |
| Q1 |  | ref |
| Q2 |  | 1.01 (0.96-1.05) |
| Q3 |  | 1.07 (1.03-1.12) |
| Q4 |  | 1.14 (1.09-1.19) |
| LMR |  |  |
| Q1 |  | ref |
| Q2 |  | 1.01 (0.97-1.04) |
| Q3 |  | 0.96 (0.92-1.00) |
| Q4 |  | 0.96 (0.91-1.00) |
| NLR |  |  |
| Q1 |  | ref |
| Q2 |  | 0.99 (0.95-1.03) |
| Q3 |  | 0.97 (0.92-1.01) |
| Q4 |  | 1.01 (0.95-1.07) |
| PLR |  |  |
| Q1 |  | ref |
| Q2 |  | 0.91 (0.88-0.95) |
| Q3 |  | 0.89 (0.85-0.92) |
| Q4 |  | 0.86 (0.82-0.91) |
| SII |  |  |
| Q1 |  | ref |
| Q2 |  | 0.95 (0.91-0.99) |
| Q3 |  | 0.92 (0.87-0.96) |
| Q4 |  | 0.89 (0.83-0.94) |
| SIRI |  |  |
| Q1 |  | ref |
| Q2 |  | 1.02 (0.97-1.06) |
| Q3 |  | 1.07 (1.03-1.12) |
| Q4 |  | 1.09 (1.03-1.14) |

CI, confidence intervals; CVD, cardiovascular disease; DBP, diastolic blood pressure; HDL, high-density lipoprotein; HR, hazard ratio; IHD, ischemic heart disease; LDL, low-density lipoprotein; LMR, Lymphocyte-to-monocyte ratio; LTCs, long-term conditions; NLR, Neutrophil-to-lymphocyte ratio; PLR, Platelet-to-lymphocyte ratio; SII, Systemic immune-inflammation index; SBP, systolic blood pressure; SD, standard deviation; TG, triglyceride; WC, waist circumference.

Model adjusted for age, sex, Townsend deprivation index, ethnicity, smoking status, weekly units of alcohol use, sleep duration, fruit and vegetable intake, processed meat intake, red meat intake, physical activity, and total sedentary time, number of long-term conditions, HDL, total cholesterol, SBP, HbA1c, BMI, and WC and the other systemic inflammation indicators when exploring one exposure.

**Supplementary Table 5.**  **Sensitivity analysis of the association of systemic inflammation indicators and incident CVD**

|  |  | **Sensitivity analysis 1** | **Sensitivity analysis 2** | **Sensitivity analysis 3** | **Sensitivity analysis 4** | **Sensitivity analysis 5** |
| --- | --- | --- | --- | --- | --- | --- |
| Outcome | **Exposure** | **HR (95% CI)** | **HR (95% CI)** | **HR (95% CI)** | **HR (95% CI)** | **HR (95% CI)** |
| **CVD** | Neutrophil count |  |  |  |  |  |
|  | Q1 | Ref. | Ref. | Ref. | Ref. | Ref. |
|  | Q2 | 1.07 (1.03-1.11) | 1.07 (1.03-1.11) | 1.07 (1.03-1.11) | 1.07 (1.03-1.11) | 1.07 (1.03-1.11) |
|  | Q3 | 1.11 (1.07-1.16) | 1.11 (1.07-1.15) | 1.11 (1.07-1.15) | 1.11 (1.07-1.15) | 1.11 (1.07-1.15) |
|  | Q4 | 1.22 (1.18-1.27) | 1.23 (1.19-1.28) | 1.23 (1.19-1.28) | 1.23 (1.18-1.27) | 1.23 (1.19-1.28) |
|  | Monocyte count |  |  |  |  |  |
|  | Q1 | Ref. | Ref. | Ref. | Ref. | Ref. |
|  | Q2 | 0.99 (0.95-1.03) | 1.00 (0.96-1.04) | 1.00 (0.96-1.04) | 1.00 (0.96-1.04) | 1.00 (0.96-1.04) |
|  | Q3 | 1.07 (1.03-1.11) | 1.07 (1.03-1.11) | 1.07 (1.04-1.12) | 1.08 (1.04-1.12) | 1.08 (1.04-1.12) |
|  | Q4 | 1.15 (1.10-1.19) | 1.14 (1.10-1.18) | 1.15 (1.11-1.19) | 1.15 (1.11-1.20) | 1.15 (1.11-1.20) |
|  | Lymphocyte count |  |  |  |  |  |
|  | Q1 | Ref. | Ref. | Ref. | Ref. | Ref. |
|  | Q2 | 1.00 (0.96-1.04) | 1.01 (0.97-1.04) | 1.01 (0.97-1.05) | 1.01 (0.97-1.05) | 1.01 (0.98-1.05) |
|  | Q3 | 0.98 (0.94-1.02) | 0.98 (0.94-1.01) | 0.98 (0.94-1.02) | 0.98 (0.94-1.02) | 0.98 (0.95-1.02) |
|  | Q4 | 1.06 (1.02-1.10) | 1.06 (1.02-1.10) | 1.07 (1.03-1.11) | 1.07 (1.03-1.11) | 1.07 (1.03-1.11) |
|  | CRP |  |  |  |  |  |
|  | Q1 | Ref. | Ref. | Ref. | Ref. | Ref. |
|  | Q2 | 1.02 (0.97-1.06) | 1.04 (0.99-1.08) | 1.03 (0.99-1.07) | 1.04 (0.99-1.08) | 1.04 (1.00-1.08) |
|  | Q3 | 1.09 (1.04-1.13) | 1.11 (1.06-1.15) | 1.10 (1.05-1.14) | 1.10 (1.06-1.14) | 1.10 (1.06-1.14) |
|  | Q4 | 1.18 (1.13-1.23) | 1.21 (1.17-1.26) | 1.19 (1.14-1.24) | 1.19 (1.15-1.24) | 1.19 (1.15-1.24) |
|  | LMR |  |  |  |  |  |
|  | Q1 | Ref. | Ref. | Ref. | Ref. | Ref. |
|  | Q2 | 0.97 (0.93-1.00) | 0.98 (0.94-1.01) | 0.97 (0.94-1.01) | 0.98 (0.94-1.01) | 0.98 (0.94-1.01) |
|  | Q3 | 0.91 (0.87-0.95) | 0.92 (0.88-0.95) | 0.92 (0.88-0.95) | 0.92 (0.88-0.95) | 0.92 (0.89-0.95) |
|  | Q4 | 0.9 (0.86-0.94) | 0.91 (0.87-0.94) | 0.91 (0.87-0.94) | 0.91 (0.87-0.94) | 0.91 (0.87-0.94) |
|  | NLR |  |  |  |  |  |
|  | Q1 | Ref. | Ref. | Ref. | Ref. | Ref. |
|  | Q2 | 1.00 (0.96-1.04) | 1.00 (0.96-1.04) | 1.01 (0.97-1.05) | 1.01 (0.96-1.04) | 1.01 (0.97-1.04) |
|  | Q3 | 1.01 (0.96-1.05) | 1.00 (0.96-1.04) | 1.00 (0.96-1.04) | 1.00 (0.96-1.04) | 1.00 (0.97-1.04) |
|  | Q4 | 1.12 (1.08-1.16) | 1.12 (1.08-1.16) | 1.12 (1.08-1.16) | 1.12 (1.08-1.16) | 1.12 (1.08-1.16) |
|  | PLR |  |  |  |  |  |
|  | Q1 | Ref. | Ref. | Ref. | Ref. | Ref. |
|  | Q2 | 0.92 (0.89-0.96) | 0.92 (0.88-0.95) | 0.92 (0.88-0.95) | 0.92 (0.88-0.95) | 0.92 (0.89-0.95) |
|  | Q3 | 0.90 (0.87-0.94) | 0.90 (0.86-0.93) | 0.9 (0.86-0.93) | 0.89 (0.86-0.93) | 0.89 (0.86-0.92) |
|  | Q4 | 0.90 (0.86-0.93) | 0.90 (0.86-0.93) | 0.9 (0.86-0.93) | 0.89 (0.86-0.92) | 0.89 (0.86-0.92) |
|  | SII |  |  |  |  |  |
|  | Q1 | Ref. | Ref. | Ref. | Ref. | Ref. |
|  | Q2 | 1.01 (0.96-1.05) | 0.98 (0.94-1.02) | 0.99 (0.95-1.02) | 0.99 (0.95-1.02) | 0.99 (0.95-1.02) |
|  | Q3 | 1.01 (0.97-1.06) | 0.98 (0.94-1.01) | 0.98 (0.94-1.02) | 0.98 (0.94-1.02) | 0.98 (0.95-1.02) |
|  | Q4 | 1.10 (1.05-1.14) | 1.03 (0.99-1.07) | 1.04 (1.00-1.08) | 1.04 (1.00-1.07) | 1.04 (1.00-1.07) |
|  | SIRI |  |  |  |  |  |
|  | Q1 | Ref. | Ref. | Ref. | Ref. | Ref. |
|  | Q2 | 1.04 (1-1.09) | 1.03 (0.99-1.07) | 1.06 (1.02-1.10) | 1.06 (1.02-1.10) | 1.04 (1.00-1.08) |
|  | Q3 | 1.12 (1.08-1.17) | 1.12 (1.07-1.16) | 1.11 (1.06-1.15) | 1.11 (1.07-1.15) | 1.12 (1.08-1.17) |
|  | Q4 | 1.21 (1.16-1.26) | 1.20 (1.16-1.25) | 1.17 (1.12-1.21) | 1.17 (1.13-1.22) | 1.21 (1.17-1.26) |
|  |  |  |  |  |  |  |
| **IHD** | Neutrophil count |  |  |  |  |  |
|  | Q1 | Ref. | Ref. | Ref. | Ref. | Ref. |
|  | Q2 | 1.09 (1.04-1.15) | 1.09 (1.04-1.13) | 1.09 (1.05-1.14) | 1.09 (1.05-1.14) | 1.09 (1.05-1.14) |
|  | Q3 | 1.11 (1.06-1.17) | 1.10 (1.05-1.14) | 1.11 (1.06-1.16) | 1.11 (1.06-1.16) | 1.11 (1.06-1.16) |
|  | Q4 | 1.18 (1.13-1.24) | 1.17 (1.12-1.22) | 1.19 (1.14-1.24) | 1.19 (1.14-1.24) | 1.19 (1.14-1.25) |
|  | Monocyte count |  |  |  |  |  |
|  | Q1 | Ref. | Ref. | Ref. | Ref. | Ref. |
|  | Q2 | 1.02 (0.96-1.07) | 1.03 (0.98-1.08) | 1.04 (0.99-1.09) | 1.04 (0.99-1.09) | 1.04 (0.99-1.09) |
|  | Q3 | 1.05 (0.99-1.09) | 1.05 (1-1.09) | 1.05 (1.01-1.10) | 1.06 (1.01-1.10) | 1.06 (1.01-1.10) |
|  | Q4 | 1.12 (1.07-1.17) | 1.12 (1.07-1.17) | 1.13 (1.08-1.18) | 1.13 (1.08-1.18) | 1.13 (1.08-1.18) |
|  | Lymphocyte count |  |  |  |  |  |
|  | Q1 | Ref. | Ref. | Ref. | Ref. | Ref. |
|  | Q2 | 0.99 (0.93-1.04) | 0.97 (0.92-1.01) | 0.98 (0.93-1.02) | 0.98 (0.93-1.02) | 0.97 (0.94-1.02) |
|  | Q3 | 1.01 (0.96-1.07) | 0.98 (0.93-1.02) | 0.99 (0.95-1.03) | 0.99 (0.94-1.03) | 0.99 (0.95-1.03) |
|  | Q4 | 1.08 (1.02-1.14) | 1.04 (0.99-1.08) | 1.05 (1.01-1.10) | 1.05 (1.01-1.10) | 1.05 (1.01-1.10) |
|  | CRP |  |  |  |  |  |
|  | Q1 | Ref. | Ref. | Ref. | Ref. | Ref. |
|  | Q2 | 1.03 (0.98-1.09) | 1.05 (0.99-1.10) | 1.04 (0.99-1.09) | 1.05 (0.99-1.10) | 1.05 (1.00-1.10) |
|  | Q3 | 1.11 (1.06-1.17) | 1.13 (1.08-1.19) | 1.12 (1.07-1.17) | 1.12 (1.07-1.17) | 1.12 (1.07-1.17) |
|  | Q4 | 1.19 (1.13-1.25) | 1.22 (1.17-1.28) | 1.19 (1.13-1.25) | 1.19 (1.13-1.25) | 1.19 (1.13-1.25) |
|  | LMR |  |  |  |  |  |
|  | Q1 | Ref. | Ref. | Ref. | Ref. | Ref. |
|  | Q2 | 0.97 (0.93-1.01) | 0.98 (0.94-1.02) | 0.98 (0.93-1.01) | 0.98 (0.94-1.02) | 0.98 (0.94-1.02) |
|  | Q3 | 0.93 (0.89-0.98) | 0.94 (0.9-0.98) | 0.94 (0.9-0.98) | 0.94 (0.9-0.98) | 0.94 (0.9-0.98) |
|  | Q4 | 0.94 (0.89-0.99) | 0.95 (0.9-0.99) | 0.95 (0.91-1) | 0.95 (0.9-0.99) | 0.95 (0.91-0.99) |
|  | NLR |  |  |  |  |  |
|  | Q1 | Ref. | Ref. | Ref. | Ref. | Ref. |
|  | Q2 | 1.02 (0.97-1.07) | 1.01 (0.97-1.06) | 1.02 (0.97-1.06) | 1.01 (0.97-1.06) | 1.01 (0.97-1.06) |
|  | Q3 | 1.00 (0.96-1.05) | 0.99 (0.95-1.04) | 0.99 (0.95-1.04) | 1.00 (0.95-1.04) | 1.00 (0.96-1.04) |
|  | Q4 | 1.11 (1.06-1.16) | 1.11 (1.06-1.15) | 1.10 (1.06-1.15) | 1.10 (1.06-1.15) | 1.11 (1.06-1.15) |
|  | PLR |  |  |  |  |  |
|  | Q1 | Ref. | Ref. | Ref. | Ref. | Ref. |
|  | Q2 | 0.96 (0.92-1.00) | 0.96 (0.92-1.00) | 0.96 (0.91-0.99) | 0.96 (0.91-0.99) | 0.96 (0.92-0.99) |
|  | Q3 | 0.96 (0.92-1.01) | 0.96 (0.91-1.00) | 0.96 (0.91-1.00) | 0.95 (0.91-0.99) | 0.95 (0.91-0.99) |
|  | Q4 | 0.95 (0.90-0.99) | 0.96 (0.91-1.00) | 0.95 (0.90-0.99) | 0.95 (0.90-0.99) | 0.95 (0.91-0.99) |
|  | SII |  |  |  |  |  |
|  | Q1 | Ref. | Ref. | Ref. | Ref. | Ref. |
|  | Q2 | 0.99 (0.93-1.04) | 0.99 (0.95-1.03) | 0.99 (0.95-1.03) | 1.00 (0.95-1.04) | 0.99 (0.95-1.04) |
|  | Q3 | 0.99 (0.94-1.05) | 1 (0.96-1.04) | 1.00 (0.96-1.05) | 1.01 (0.96-1.05) | 1.01 (0.97-1.05) |
|  | Q4 | 1.04 (0.98-1.09) | 1.06 (1.02-1.11) | 1.06 (1.02-1.11) | 1.06 (1.02-1.11) | 1.07 (1.02-1.11) |
|  | SIRI |  |  |  |  |  |
|  | Q1 | Ref. | Ref. | Ref. | Ref. | Ref. |
|  | Q2 | 1.03 (0.98-1.08) | 1.02 (0.97-1.06) | 1.06 (1.01-1.11) | 1.06 (1.01-1.11) | 1.02 (0.97-1.07) |
|  | Q3 | 1.09 (1.04-1.15) | 1.08 (1.04-1.13) | 1.05 (0.99-1.09) | 1.04 (0.99-1.09) | 1.09 (1.04-1.14) |
|  | Q4 | 1.15 (1.09-1.20) | 1.14 (1.09-1.19) | 1.11 (1.06-1.16) | 1.12 (1.07-1.17) | 1.15 (1.10-1.20) |
| **Stroke** | Neutrophil count |  |  |  |  |  |
|  | Q1 | Ref. | Ref. | Ref. | Ref. | Ref. |
|  | Q2 | 0.96 (0.88-1.04) | 0.96 (0.88-1.04) | 0.96 (0.88-1.04) | 0.96 (0.88-1.04) | 0.96 (0.89-1.04) |
|  | Q3 | 0.97 (0.89-1.05) | 0.97 (0.89-1.05) | 0.97 (0.89-1.05) | 0.97 (0.89-1.05) | 0.97 (0.90-1.05) |
|  | Q4 | 1.09 (1.00-1.18) | 1.09 (1.00-1.17) | 1.08 (0.99-1.17) | 1.09 (1.01-1.18) | 1.09 (1.01-1.18) |
|  | Monocyte count |  |  |  |  |  |
|  | Q1 | Ref. | Ref. | Ref. | Ref. | Ref. |
|  | Q2 | 1.01 (0.92-1.10) | 1.01 (0.93-1.10) | 1.01 (0.93-1.10) | 1.01 (0.93-1.10) | 1.01 (0.93-1.10) |
|  | Q3 | 1.06 (0.97-1.16) | 1.07 (0.98-1.16) | 1.07 (0.98-1.16) | 1.07 (0.98-1.16) | 1.07 (0.99-1.16) |
|  | Q4 | 1.15 (1.05-1.24) | 1.15 (1.06-1.25) | 1.15 (1.06-1.25) | 1.15 (1.07-1.25) | 1.15 (1.07-1.25) |
|  | Lymphocyte count |  |  |  |  |  |
|  | Q1 | Ref. | Ref. | Ref. | Ref. | Ref. |
|  | Q2 | 0.94 (0.86-1.01) | 0.95 (0.87-1.02) | 0.95 (0.88-1.03) | 0.95 (0.87-1.02) | 0.95 (0.88-1.02) |
|  | Q3 | 0.92 (0.85-1.00) | 0.92 (0.84-0.99) | 0.92 (0.84-0.99) | 0.92 (0.84-0.99) | 0.91 (0.85-0.99) |
|  | Q4 | 1.01 (0.93-1.10) | 1.02 (0.94-1.10) | 1.03 (0.94-1.11) | 1.02 (0.94-1.10) | 1.02 (0.94-1.10) |
|  | CRP |  |  |  |  |  |
|  | Q1 | Ref. | Ref. | Ref. | Ref. | Ref. |
|  | Q2 | 0.96 (0.88-1.05) | 0.99 (0.90-1.07) | 0.98 (0.90-1.07) | 0.99 (0.91-1.07) | 0.99 (0.91-1.07) |
|  | Q3 | 0.99 (0.91-1.08) | 1.00 (0.91-1.08) | 0.99 (0.91-1.08) | 1.00 (0.91-1.08) | 1.00 (0.92-1.08) |
|  | Q4 | 1.11 (1.02-1.22) | 1.14 (1.05-1.24) | 1.13 (1.03-1.23) | 1.14 (1.05-1.24) | 1.14 (1.05-1.25) |
|  | LMR |  |  |  |  |  |
|  | Q1 | Ref. | Ref. | Ref. | Ref. | Ref. |
|  | Q2 | 0.94 (0.86-1.01) | 0.95 (0.88-1.02) | 0.96 (0.89-1.03) | 0.95 (0.88-1.02) | 0.95 (0.88-1.02) |
|  | Q3 | 0.89 (0.82-0.97) | 0.89 (0.82-0.96) | 0.9 (0.83-0.97) | 0.89 (0.82-0.96) | 0.89 (0.82-0.96) |
|  | Q4 | 0.9 (0.82-0.98) | 0.9 (0.82-0.97) | 0.9 (0.83-0.98) | 0.89 (0.82-0.97) | 0.89 (0.82-0.97) |
|  | NLR |  |  |  |  |  |
|  | Q1 | Ref. | Ref. | Ref. | Ref. | Ref. |
|  | Q2 | 0.95 (0.87-1.03) | 0.95 (0.88-1.03) | 0.96 (0.88-1.04) | 0.96 (0.88-1.03) | 0.96 (0.88-1.03) |
|  | Q3 | 0.99 (0.91-1.08) | 0.99 (0.91-1.07) | 0.98 (0.90-1.06) | 0.99 (0.91-1.07) | 0.99 (0.91-1.07) |
|  | Q4 | 1.04 (0.96-1.13) | 1.05 (0.97-1.14) | 1.04 (0.96-1.13) | 1.06 (0.97-1.14) | 1.06 (0.98-1.14) |
|  | PLR |  |  |  |  |  |
|  | Q1 | Ref. | Ref. | Ref. | Ref. | Ref. |
|  | Q2 | 0.93 (0.86-1.01) | 0.95 (0.88-1.03) | 0.95 (0.88-1.02) | 0.95 (0.88-1.03) | 0.95 (0.88-1.03) |
|  | Q3 | 0.93 (0.85-1.01) | 0.94 (0.86-1.01) | 0.94 (0.86-1.01) | 0.94 (0.87-1.01) | 0.94 (0.87-1.01) |
|  | Q4 | 1.05 (0.96-1.13) | 1.06 (0.98-1.14) | 1.05 (0.97-1.14) | 1.06 (0.98-1.14) | 1.06 (0.98-1.14) |
|  | SII |  |  |  |  |  |
|  | Q1 | Ref. | Ref. | Ref. | Ref. | Ref. |
|  | Q2 | 1.09 (0.97-1.21) | 1.02 (0.94-1.10) | 1.03 (0.94-1.11) | 1.02 (0.94-1.10) | 1.02 (0.94-1.10) |
|  | Q3 | 1.07 (0.95-1.19) | 1.03 (0.95-1.11) | 1.03 (0.95-1.12) | 1.03 (0.95-1.11) | 1.03 (0.95-1.11) |
|  | Q4 | 1.13 (1.02-1.26) | 1.10 (1.02-1.18) | 1.10 (1.02-1.19) | 1.10 (1.02-1.19) | 1.10 (1.02-1.19) |
|  | SIRI |  |  |  |  |  |
|  | Q1 | Ref. | Ref. | Ref. | Ref. | Ref. |
|  | Q2 | 0.96 (0.87-1.05) | 0.98 (0.89-1.06) | 0.97 (0.88-1.05) | 0.97 (0.89-1.06) | 0.98 (0.90-1.06) |
|  | Q3 | 1.05 (0.96-1.14) | 1.06 (0.97-1.15) | 1.10 (1.01-1.19) | 1.10 (1.01-1.19) | 1.06 (0.98-1.15) |
|  |  | 1.14 (1.04-1.24) | 1.15 (1.06-1.24) | 1.13 (1.04-1.23) | 1.14 (1.05-1.24) | 1.15 (1.06-1.25) |
| **Heart failure** | Neutrophil count |  |  |  |  |  |
|  | Q1 | Ref. | Ref. | Ref. | Ref. | Ref. |
|  | Q2 | 1.05 (0.96-1.13) | 1.06 (0.97-1.14) | 1.05 (0.97-1.14) | 1.06 (0.98-1.14) | 1.06 (0.98-1.15) |
|  | Q3 | 1.10 (1.02-1.19) | 1.11 (1.03-1.20) | 1.11 (1.03-1.20) | 1.11 (1.03-1.20) | 1.11 (1.03-1.20) |
|  | Q4 | 1.29 (1.20-1.40) | 1.32 (1.23-1.42) | 1.32 (1.23-1.42) | 1.33 (1.24-1.43) | 1.33 (1.24-1.43) |
|  | Monocyte count |  |  |  |  |  |
|  | Q1 | Ref. | Ref. | Ref. | Ref. | Ref. |
|  | Q2 | 0.99 (0.91-1.07) | 1.00 (0.92-1.08) | 1.00 (0.91-1.08) | 1.00 (0.92-1.08) | 1.00 (0.93-1.08) |
|  | Q3 | 1.02 (0.94-1.10) | 1.01 (0.94-1.09) | 1.02 (0.94-1.10) | 1.02 (0.94-1.10) | 1.02 (0.95-1.10) |
|  | Q4 | 1.15 (1.07-1.24) | 1.16 (1.08-1.25) | 1.17 (1.08-1.25) | 1.17 (1.09-1.26) | 1.17 (1.09-1.26) |
|  | Lymphocyte count |  |  |  |  |  |
|  | Q1 | Ref. | Ref. | Ref. | Ref. | Ref. |
|  | Q2 | 0.84 (0.78-0.91) | 0.86 (0.79-0.92) | 0.86 (0.8-0.92) | 0.86 (0.8-0.92) | 0.86 (0.80-0.92) |
|  | Q3 | 0.80 (0.74-0.86) | 0.80 (0.74-0.86) | 0.8 (0.75-0.86) | 0.80 (0.74-0.86) | 0.80 (0.75-0.86) |
|  | Q4 | 0.84 (0.78-0.9) | 0.85 (0.79-0.91) | 0.85 (0.79-0.91) | 0.85 (0.79-0.91) | 0.85 (0.80-0.91) |
|  | CRP |  |  |  |  |  |
|  | Q1 | Ref. | Ref. | Ref. | Ref. | Ref. |
|  | Q2 | 1.03 (0.94-1.12) | 1.05 (0.96-1.14) | 1.05 (0.96-1.14) | 1.05 (0.97-1.14) | 1.05 (0.97-1.14) |
|  | Q3 | 1.14 (1.05-1.24) | 1.17 (1.08-1.27) | 1.17 (1.08-1.27) | 1.17 (1.08-1.27) | 1.17 (1.08-1.27) |
|  | Q4 | 1.38 (1.27-1.50) | 1.42 (1.31-1.54) | 1.42 (1.31-1.54) | 1.42 (1.31-1.54) | 1.43 (1.32-1.55) |
|  | LMR |  |  |  |  |  |
|  | Q1 | Ref. | Ref. | Ref. | Ref. | Ref. |
|  | Q2 | 0.85 (0.79-0.91) | 0.86 (0.81-0.92) | 0.85 (0.80-0.91) | 0.86 (0.8-0.92) | 0.86 (0.81-0.92) |
|  | Q3 | 0.78 (0.72-0.84) | 0.77 (0.72-0.83) | 0.76 (0.71-0.82) | 0.77 (0.71-0.82) | 0.77 (0.72-0.82) |
|  | Q4 | 0.75 (0.69-0.81) | 0.76 (0.70-0.81) | 0.75 (0.69-0.81) | 0.75 (0.69-0.81) | 0.75 (0.70-0.81) |
|  | NLR |  |  |  |  |  |
|  | Q1 | Ref. | Ref. | Ref. | Ref. | Ref. |
|  | Q2 | 1.08 (0.99-1.16) | 1.09 (1.01-1.18) | 1.11 (1.02-1.19) | 1.09 (1.01-1.18) | 1.09 (1.01-1.18) |
|  | Q3 | 1.11 (1.02-1.20) | 1.13 (1.05-1.21) | 1.13 (1.05-1.22) | 1.13 (1.05-1.22) | 1.13 (1.05-1.22) |
|  | Q4 | 1.43 (1.33-1.54) | 1.45 (1.35-1.56) | 1.46 (1.36-1.57) | 1.46 (1.36-1.56) | 1.46 (1.36-1.56) |
|  | PLR |  |  |  |  |  |
|  | Q1 | Ref. | Ref. | Ref. | Ref. | Ref. |
|  | Q2 | 0.98 (0.91-1.05) | 0.96 (0.90-1.03) | 0.96 (0.89-1.03) | 0.96 (0.90-1.03) | 0.96 (0.90-1.03) |
|  | Q3 | 1.01 (0.94-1.08) | 1.00 (0.93-1.07) | 1.00 (0.93-1.07) | 1.00 (0.93-1.07) | 1.00 (0.93-1.07) |
|  | Q4 | 1.13 (1.06-1.22) | 1.12 (1.05-1.20) | 1.13 (1.06-1.21) | 1.12 (1.05-1.20) | 1.12 (1.05-1.20) |
|  | SII |  |  |  |  |  |
|  | Q1 | Ref. | Ref. | Ref. | Ref. | Ref. |
|  | Q2 | 0.99 (0.89-1.09) | 1.02 (0.94-1.09) | 1.01 (0.94-1.09) | 1.02 (0.94-1.09) | 1.02 (0.95-1.09) |
|  | Q3 | 1.04 (0.94-1.15) | 1.06 (0.99-1.14) | 1.06 (0.98-1.14) | 1.07 (0.99-1.14) | 1.07 (0.99-1.14) |
|  | Q4 | 1.29 (1.17-1.41) | 1.27 (1.19-1.36) | 1.28 (1.20-1.37) | 1.28 (1.19-1.37) | 1.28 (1.20-1.37) |
|  | SIRI |  |  |  |  |  |
|  | Q1 | Ref. | Ref. | Ref. | Ref. | Ref. |
|  | Q2 | 1.11 (1.02-1.21) | 1.10 (1.01-1.19) | 1.04 (0.95-1.13) | 1.04 (0.96-1.13) | 1.10 (1.01-1.20) |
|  | Q3 | 1.19 (1.10-1.29) | 1.19 (1.10-1.29) | 1.11 (1.03-1.20) | 1.12 (1.04-1.21) | 1.20 (1.11-1.30) |
|  | Q4 | 1.47 (1.36-1.59) | 1.48 (1.37-1.60) | 1.29 (1.20-1.39) | 1.30 (1.20-1.40) | 1.50 (1.39-1.62) |

CI, confidence intervals; HDL, high-density lipoprotein; HR, hazard ratio; IHD, ischemic heart disease; LDL, low-density lipoprotein; LMR, Lymphocyte-to-monocyte ratio; LTCs, long-term conditions; NLR, Neutrophil-to-lymphocyte ratio; PLR, Platelet-to-lymphocyte ratio; SII, Systemic immune-inflammation index; SBP, systolic blood pressure; WC, waist circumference.

Model adjusted for age, sex, Townsend deprivation index, ethnicity, smoking status, weekly units of alcohol use, sleep duration, fruit and vegetable intake, processed meat intake, red meat intake, physical activity, and total sedentary time, number of LTCs, HDL, total cholesterol, SBP, HbA1c, BMI, and WC. In sensitivity analysis 2, the model additionally adjusted for insulin, and antihypertensive and cholesterol-lowering medications; in sensitivity analysis 4, adjusting for Downtown deprivation index as continuous variable instead of categorical variable.


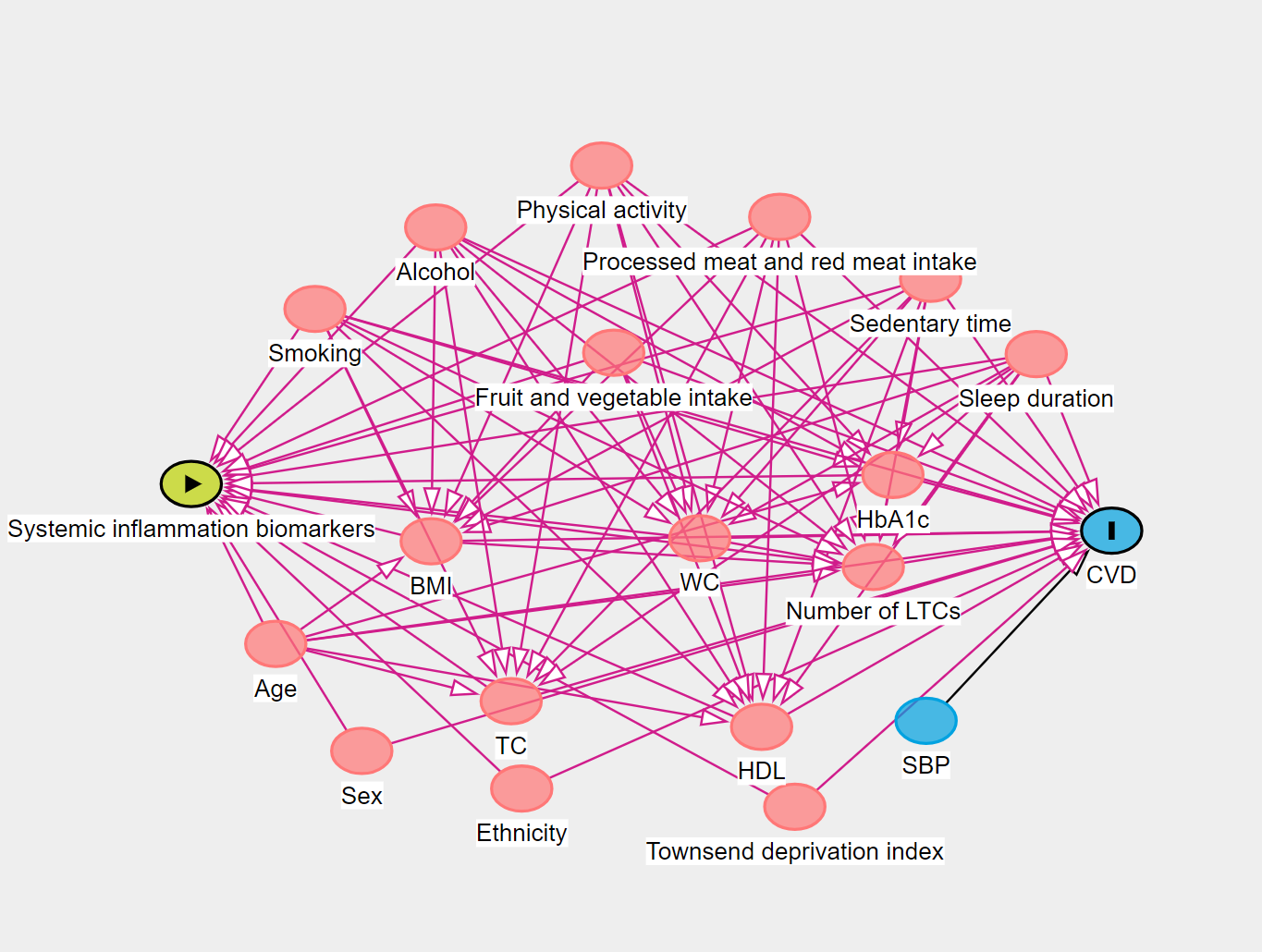


**Supplemental Figure 1. Directed acyclic graphs (DAGs) depicting the assumed underlying causal paths between systemic inflammation biomarkers and incident cardiovascular disease.**

Figure created using DAGitty (http://dagitty.net/): Johannes Textor, Benito van der Zander, Mark K. Gilthorpe, Maciej Liskiewicz, George T.H. Ellison. Robust causal inference using directed acyclic graphs: the R package 'dagitty'. International Journal of Epidemiology 45(6):1887-1894, 2016.


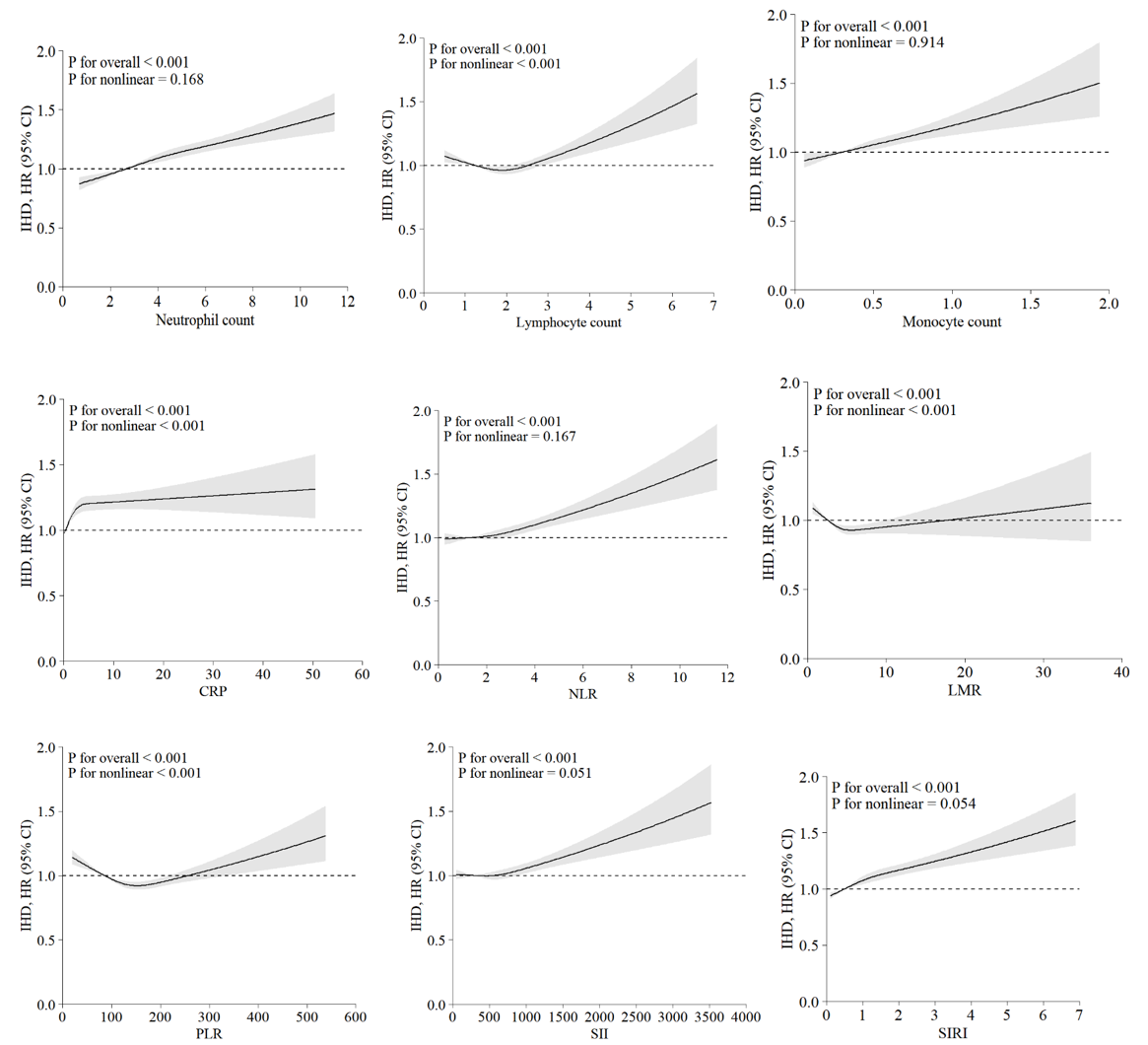


**Supplementary Figure 2 Dose-response associations of chronic systemic inflammation biomarkers and incident ischemic heart disease.**

Restricted cubic splines were used to present the associations. The model was adjusted for age, sex, Townsend deprivation index, ethnicity, smoking status, weekly units of alcohol use, sleep duration, fruit and vegetable intake, processed meat intake, red meat intake, physical activity, total sedentary time, number of LTCs, HDL, total cholesterol, SBP, HbA1c, BMI, and WC. BMI, body mass index; CI, confidence interval; HDL, high-density lipoprotein cholesterol; HR, hazard ratio; IHD, ischemic heart disease; LMR, Lymphocyte-to-monocyte ratio; LTCs, long-term conditions; NLR, Neutrophil-to-lymphocyte ratio; PLR, Platelet-to-lymphocyte ratio; SII, Systemic immune-inflammation index; SIRI, system inflammation response index; SBP, systolic blood pressure; WC, waist circumference.


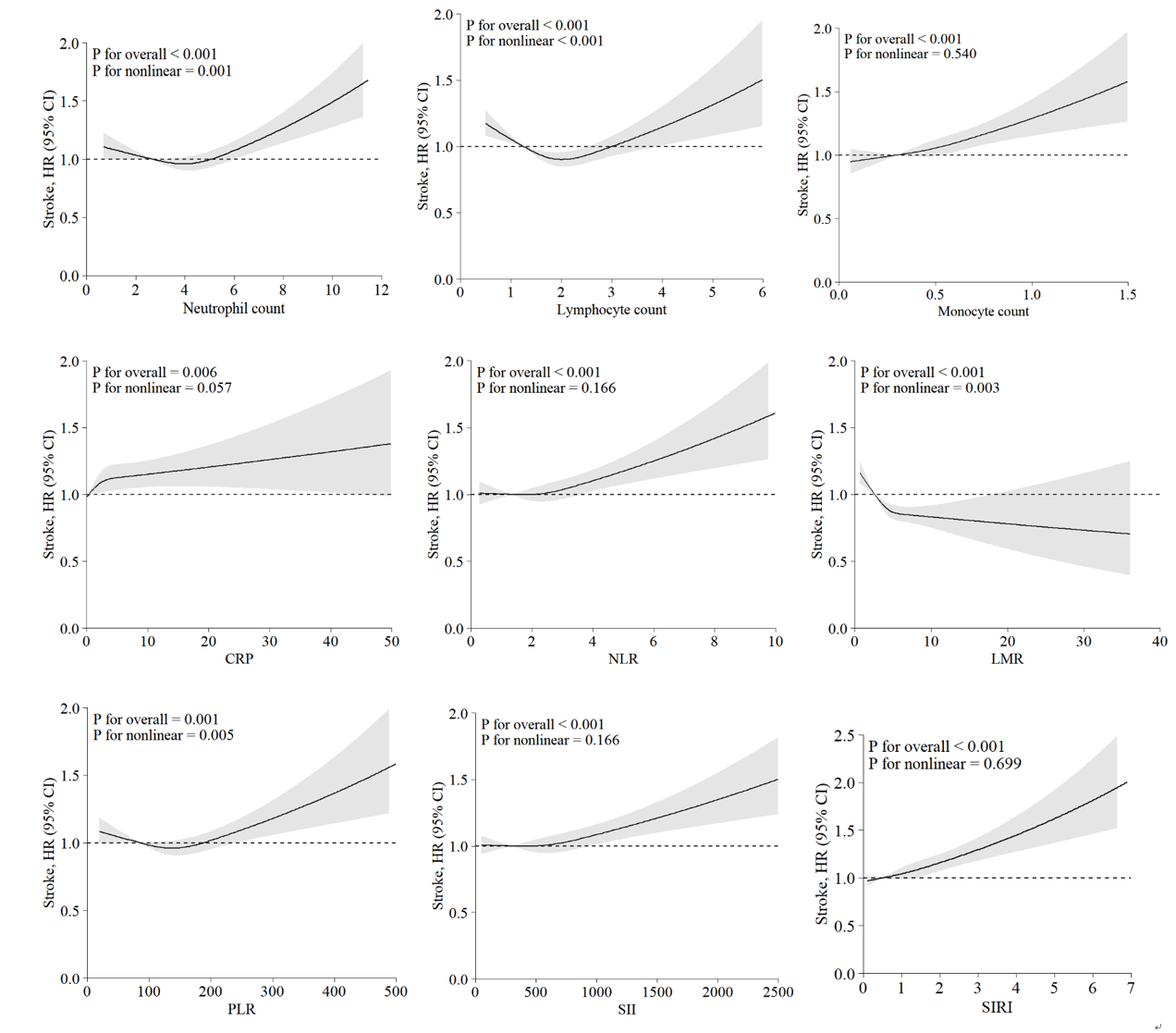


**Supplementary Figure 3 Dose-response associations of chronic systemic inflammation biomarkers and incident stroke.**

Restricted cubic splines were used to present the associations. The model was adjusted for age, sex, Townsend deprivation index, ethnicity, smoking status, weekly units of alcohol use, sleep duration, fruit and vegetable intake, processed meat intake, red meat intake, physical activity, total sedentary time, number of LTCs, HDL, total cholesterol, SBP, HbA1c, BMI, and WC. BMI, body mass index; CI, confidence interval; HDL, high-density lipoprotein cholesterol; HR, hazard ratio; LMR, Lymphocyte-to-monocyte ratio; LTCs, long-term conditions; NLR, Neutrophil-to-lymphocyte ratio; PLR, Platelet-to-lymphocyte ratio; SBP, systolic blood pressure; SII, Systemic immune-inflammation index; SIRI, system inflammation response index; WC, waist circumference.


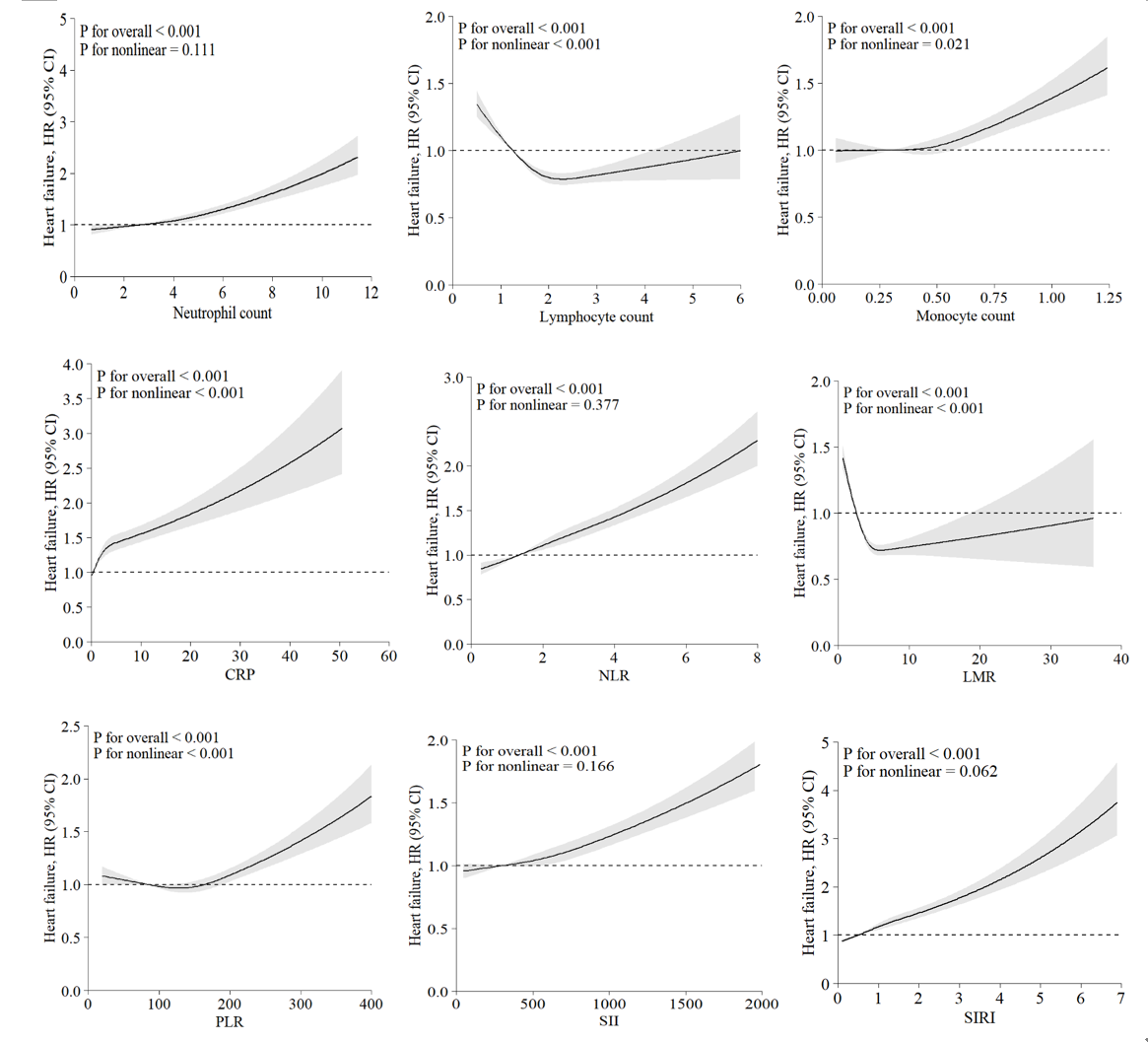


**Supplementary Figure 4 Dose-response associations of chronic systemic inflammation biomarkers and incident heart failure.**

Restricted cubic splines were used to present the associations. The model was adjusted for age, sex, Townsend deprivation index, ethnicity, smoking status, weekly units of alcohol use, sleep duration, fruit and vegetable intake, processed meat intake, red meat intake, physical activity, total sedentary time, number of LTCs, HDL, total cholesterol, SBP, HbA1c, BMI, and WC. BMI, body mass index; CI, confidence interval; HDL, high-density lipoprotein cholesterol; HR, hazard ratio; LMR, Lymphocyte-to-monocyte ratio; LTCs, long-term conditions; NLR, Neutrophil-to-lymphocyte ratio; PLR, Platelet-to-lymphocyte ratio; SBP, systolic blood pressure; SII, Systemic immune-inflammation index; SIRI, system inflammation response index; WC, waist circumference.
